# A novel strategy for process optimization of a natural gas liquid recovery unit by replacing Joule–Thomson valve with supersonic separator

**DOI:** 10.1038/s41598-022-26692-z

**Published:** 2022-12-27

**Authors:** Sina Nabati Shoghl, Abbas Naderifar, Fatola Farhadi, Gholamreza Pazuki

**Affiliations:** 1 Department of Chemical Engineering, Amirkabir University of Technology (Tehran Polytechnic), Tehran, Iran; 2 Department of Chemical and Petroleum Engineering, Sharif University of Technology, Azadi Ave., Tehran, Iran

**Keywords:** Chemical engineering, Mechanical engineering

## Abstract

The natural gas liquid recovery is an important process in a gas plant to correct hydrocarbon dew point and earn profit. In this study, a natural gas liquid recovery unit operated based on the Joule–Thomson process was investigated and its performance was optimized. To improve the system performance, the plant configuration and intermediate pressure ratio were defined as the variables and maximization of the natural gas liquid recovery rate and maximization of exergy efficiency were defined as the objective functions. To improve the plant performance, the amount of natural gas liquid recovery rate should be increased. To achieve this goal, several scenarios for the intermediate pressure ratio and three new configurations were proposed for the investigated gas plant. In the proposed configurations, the supersonic separators with optimized structures were used instead of the Joule–Thomson process. It was observed that all three proposed configurations improved the natural gas liquid recovery rate compared to the existing configuration. For example, by installing two supersonic separators instead of second and third stage Joule–Thomson valve + low temperature separator, at the optimal operating condition, the natural gas liquid recovery rate increased about 390%. The influence of the intermediate pressure ratio on the phase envelope diagram, exergy efficiency, dew point depression and natural gas liquid recovery rate was also investigated. By comparing the influence of intermediate pressure ratio and modifying the plant configuration on the objective functions, it was observed that the system performance can be further improved by modifying the plant configuration.

## Introduction

The produced natural gas from wells is saturated with heavy hydrocarbons (HCs) and water vapor. The process of separation of heavy HCs from natural gas stream is called natural gas liquid (NGL) recovery. The cryogenic processes are the most common method of NGL recovery in the natural gas industry^1–3^. The number of the NGL recovery plants are growing in the last years due to the increasing demand for this product^4^. Due to the high price of natural gas condensate as well as the necessity of correcting the natural gas dew point, the NGL recovery is set up. The NGL recovery can be conducted in numerous methods such as using cryogenic refrigeration, Joule–Thomson (JT) process, turbo-expander, vortex tube and supersonic separator (3S)^5,6^. The conventional separation methods like the JT process require large equipment, high operating and capital cost, large pressure drop for a specific separation and negative influence on the environment due to the injection of chemical inhibitors. The novel 3S overcomes these deficiencies^7–9^. The compact design and simple configuration of the 3S make it suitable for off-shore plant and unmanned operation. One of the questions that is always raised is, what are the advantages and disadvantages of using 3S instead of conventional processes such as the JT process in a gas plant. It was observed that, the 3S has a good cooling performance compared to the other self-cooling systems like turbo-expander and JT process^5^. The first engineering team focused on this technology was a group from Twister BV^10^. Another Russian engineering team^11^ also worked on this separator. This technology has attracted considerable attention in the Oil & Gas industry.

In order to replace the 3S with conventional separation processes, the structure of it should be optimized first. These optimizations can include the optimization of the profile of the wall, swirler structures and the dimensions of a 3S^12,13^. In the last decade, some researchers focused on the optimization of the structure and the separation performance of the 3S. For example, Jiang and Bian et al.^14–18^ employed the discrete particle method and the field experiment for the optimization of the 3S. They increased the length of the expanding section and decreased the expanding angle to improve the separation performance. Wen et al.^19–21^ optimized the structure of swirler and diffuser. Liu et al.^22–24^ optimized the separation performance of the 3S using the flow properties and the relationship between the shockwave position and pressure effect. Jassim et al.^25,26^ evaluated the influence of several parameters including vorticity, nozzle structure and real gas properties on the performance of the 3S using the computational fluid dynamics (CFD) modeling. They observed that the shockwave location varied considerably when the natural gas state assumed real rather than perfect. Wen et al.^27,28^ investigated the 3S both numerically and experimentally and reported that installing the inner body maintained the conservation of angular momentum. Yang et al.^29^ studied the effect of a primary nozzle on the performance of steam ejector taking into account the phase change process. They reported that the first non-equilibrium condensation occurred within the primary nozzle, while second nucleation condensation occurred in the steam ejector. Yang et al.^30^ developed a wet steam model using CFD analysis to investigate the intricate feature of the steam condensation in the supersonic ejector. They reported the expansion feature of the primary nozzle was exaggerated by the dry gas model compared to the wet stream model. Furthermore, they observed that the dry gas model over-estimated a higher entrainment ratio by 11.71% compared to the wet steam model. Wen et al.^31^ developed the single-phase and two-phase model and analyzed the performance of steam ejector. The result of their analysis showed that a single phase flow model with pass over the phase change provided an un-physical steam temperature through the supersonic flow. Liu et al.^32^ employed the Discrete particle method to predict droplet behavior inside the 3S. They assumed that the droplet diameter varied from 10 to 50 µm, while the proper droplet diameter in the 3S was about 0.1–2 µm^33^. Wen et al.^19^ investigated the influence of different structural parameters of diffuser on the shockwave position and pressure recovery performance. They reported that for natural gas dehydration, the conical diffuser showed the best pressure recovery performance. Wen et al.^34^ investigated the influence of three delta wings with different sizes on the natural gas swirling flow. They reported that for 2 µm droplets, a collection efficiency of 70% can be obtained for the large delta wing.

Operational condition and process configuration are two main factors which can influence the efficiency of an NGL recovery plant^35,36^. Furthermore, in a gas plant, the employed liquefaction process plays a crucial role^37^ on the system performance. For example, Arinelli^7^ compared the 3S capability with conventional separation methods for HC and water dew pointing of CO_2_ rich humid natural gas. They observed that the 3S performance for HC and water dew point adjustment was higher than conventional separation methods such as the JT process. Wen et al.^38^ suggested a different method for carbon dioxide capture using supersonic flow. They developed a CFD model to describe the carbon dioxide condensation in a supersonic nozzle. A comparative investigation was conducted to analyze the influence of carbon dioxide condensation by the dry gas assumption and condensation model. They observed that the developed CFD model predicts accurately the temperature distribution opposite to the dry gas assumption. Ding et al.^39^ proposed a new Eulerian–Lagrangian method coupled with the Eulerian wall film model to investigate the phase change and three-field behaviors to improve the separation efficiency through the 3S. They verified their model using three experiments. The results of their analysis demonstrated that the separation efficiency can be improved by proper inlet pressure and inlet droplet diameter. Alferov et al.^11^ studied the natural gas separation performance using the 3S and compared this separation method with the JT process and turbo-expander. They reported that at the same differential pressure, the 3S provided significantly lower operating temperature in the liquid separation section. Wen et al.^40^ suggested a novel concept to separate carbon dioxide from the offshore natural gas industry which employed the combined influence from non-equilibrium condensation phenomena. The results of their analysis using the real gas model showed that supersonic flow can liquefy 28% carbon dioxide from the main gas flow. The ideal gas model under-predicted the liquid fraction by 46% in comparison to the real gas model. Ding et al.^41^ proposed a method to separate carbon dioxide by the phase change through the supersonic flow. They compared the accuracy of the real gas model and the ideal gas model and reported the real gas model provided a more accurate prediction of carbon dioxide condensation through the supersonic flow. Machado et al.^42^ compared technically and economically the Twister supersonic separation technology with conventional separation methods like the TEG Dehydration unit coupled to the JT process. They reported that this novel method (3S) enhanced the NGL recovery rate which provided additional revenue. Wen et al.^43^ studied a high pressure supersonic flow to analyze the natural gas dehydration potential by non-equilibrium condensation. They used a CFD model to investigate the nanodroplets formation during the phase change process. They observed that ideal gas modeling concluded the earlier onset of non-equilibrium condensation. Furthermore, the ideal gas modeling under-predicted the fraction of liquid by 61% compared to the real gas model. Wen et al.^44^ compared the phase envelope diagram (PED) derived by HYSYS simulation software and pressure and temperature profile derived by FLUENT software and reported that the 3S was appropriate for liquefaction of natural gas. Wen et al.^45^ reported that due to the complicated feature of supersonic flow, the fluid flow, heat and mass transfer in a 3S were not realized completely. Therefore, a wet steam model was developed by them to study the flow structure through the 3S. The results of their analysis showed that water vapor super-saturation can enhance to a value of 4.28 within designed 3S and provided a maximum nucleation rate of 10^21^ kg m^−3^ s^−1^. Fahmy et al.^46^ improved the performance of a liquefaction process, by replacing the JT valve with expanders. As can be seen, by modifying the structure of a gas plant, the NGL recovery rate can be improved significantly^7,44^. For example, employing a 3S instead of common processes such as the JT process and turbo-expander significantly improves the NGL recovery rate^5,42^. Vatani et al.^47^ reported that the overall energy efficiency of Liquefied natural gas (LNG) processes cannot significantly improve by structural optimization. There are numerous operational and structural parameters which affect the NGL recovery rate and exergy destruction through the system. For instance, Park et al.^48^ proposed a new configuration for an NGL recovery unit and compared it with nine different processes for NGL recovery. They reported that based on the capital cost and heat integration, the highest efficiency was obtained for the proposed configuration. Vatani et al.^49^ proposed a new configuration with lower energy consumption for producing both LNG and NGL. Therefore, in addition to the amount of NGL recovery rate, the best process should also have a higher exergy efficiency^50–52^. Therefore, in this article, the most appropriate structure and operating conditions that provide the highest amount of NGL recovery rate with the lowest energy consumption are investigated.

The conventional separation methods for NGL recovery operated based on the refrigeration system. One option is to integrate these separation processes simultaneously. For example, Cuellar et al.^53^ observed that the capital and operating cost could significantly decrease using an integrated NGL/LNG co-production design. Vatani et al.^49^ investigated and analyzed the capital cost of an integrated NGL/LNG configuration. They observed that significant condensation efficiency and high ethane recovery can be obtained for a rich feed composition. Wang^54^ investigated a turbo-expansion plant to find the optimal operating condition for various feed compositions. He observed that the minimum exergy requirement was obtained for a combination of mechanical refrigeration and turbo-expansion. Mehrpooya et al.^55^ analyzed three integrated refrigeration processes including dual mixed refrigerant, propane precooled mixed refrigerant and mixed fluid cascade. They observed that these refrigeration systems have lower power consumption and high ethane recovery compared to the previously considered process. An integrated NGL recovery for natural gas liquefaction was studied by Wang and Abbas^56^. They observed that the integrated process showed an increase of 0.74% in energy consumption, whereas a decrease of 0.18% of the same was observed for a front-end NGL recovery plant. In past studies, they were generally focused on the comparison between different processes such as 3S, JT and turbo-expander^50,52,57^. For example, Okimoto et al.^57^ compared three different processes including JT, Turbo-expander and Twister 3S, separately. They reported that as long as all three processes have the same outlet pressure, the JT process is the least efficient. Two other processes, including turbo-spender and 3S, showed 85 and 90% isentropic efficiency. Interlenghi et al.^52^ compared the performance of 3S with two different adiabatic expansion processes including JT process and turbo-expander process. These processes all start from a condition of 298.15 K and 80 bar to saturated liquefied natural gas at 115.4 K and 1.25 bar. They reported that the 3S provided the highest net present values of all routes but based on the lost power, thermodynamic efficiency and power consumption was somewhat outperformed by turbo-expander. While one option is to use a specific type of process equipment, another option is to use these equipment in an integrated manner. Therefore, while it was reported that the use of a 3S instead of conventional processes improves the efficiency of a gas plant^5,52^, an option is to use an integrated configuration. This structure includes the simultaneous use of these two processes. In these structures, heavy HCs are separated in the conventional processes like JT process^51^ and lighter HCs can also be recovered in the 3S^6^. Therefore, in a gas plant, with the help of the integration process, it will be possible to recover both heavy and light hydrocarbons.

The separation of heavy HCs from natural gas is a crucial process to correct HC dew point (HCDP) and earn profit. To increase the gas plant efficiency, the NGL recovery rate should be increased. Modification of design parameters along with process optimization is a familiar way of improving the performance of a gas plant. To the best of the author’s knowledge, the existing literature on the optimization of an NGL recovery gas plant configuration using the 3S is thin. Most past research has focused exclusively on the role of the 3S. While it is necessary, the role of the 3S in a gas plant be investigated considering different structures and operating conditions. One of these structures that needs to be investigated is the integrated structure of the JT process and the 3S. For this purpose, a gas plant that operates based on the JT process was selected as a case study. In the investigated gas plant, the energy consumption is high and NGL recovery rate is low due to the non-optimal operating condition and process scheme. Therefore, the NGL recovery rate and exergy efficiency were considered as the objective functions. In addition, the intermediate pressure ratio and gas plant configuration were defined as the variables. The main goal of this research is to propose a new configuration for recovery of natural gas condensate using the 3S or integrated 3S with the JT process at the optimal operating condition. In order to gain a deeper understanding of the behavior of the 3S, the effect of using two different droplet sizes was studied. On this basis, considering the effect of entrainment, the phase behavior of outlet gas was fully investigated. On the other hand, with the help of examining the phase behavior of the outlet gas, we seek to answer the question whether the more the dew point depression is, the more the NGL recovery is or not?. The results of this paper can be employed for the process optimization and selecting suitable configuration for NGL recovery by cryogenic processes.

## Mathematical modeling

### CFD modeling

The HCDP correction and separation of NGL from the natural gas stream are the main objective of the investigated gas plant. Like the JT process, the 3S employed the natural gas expansion to provide the partial condensation in the natural gas stream. The conservation equation of mass, momentum and energy were used to describe the natural gas flow through the 3S. The conservation equation of mass is defined as:

1 $$ \frac{\partial }{\partial t} + \frac{\partial }{{\partial x_{i} }}\left( {\rho u_{i} } \right) = 0 $$

The conservation equation of momentum is defined as:

2 $$ \frac{\partial }{\partial t}\left( {\rho u_{i} } \right) + \frac{\partial }{{\partial x_{j} }}\left( {\rho u_{i} u_{j} } \right) + \frac{\partial p}{{\partial x_{i} }} - \frac{{\partial \tau_{ij} }}{{\partial x_{j} }} - \frac{{\partial \tau_{ij}^{ - 1} }}{{\partial x_{j} }} = 0 $$

The energy equation is described as:

3 $$ \rho C_{p} \frac{DT}{{Dt}} = k\nabla^{2} T + Q_{p} + Q + \mu \phi_{v} $$

where *p, C*_*p*_*, T, µ, t, ρ, τ, Q*_*p*_*, ϕ*_*v*_*, u* and *Q* are pressure, specific heat capacity, temperature, dynamic viscosity, time, density, viscous stress tensor, pressure work, viscous dissipation, velocity and heat flux, respectively. The pressure work (*Q*_*p*_) and viscous dissipation (*ϕ*_*v*_) are calculated by Eqs. (4) and (5), respectively.

4 $$ Q_{P} = - \frac{Tu}{\rho }\left( {\frac{\partial \rho }{{\partial T}}} \right)_{P} .\nabla P_{A} $$

5 $$ \begin{gathered} \phi_{v} = 2\left[ {\left( {\frac{{\partial u_{x} }}{\partial x}} \right)^{2} + \left( {\frac{{\partial u_{y} }}{\partial y}} \right)^{2} + \left( {\frac{{\partial u_{z} }}{\partial z}} \right)^{2} } \right] + \left[ {\left( {\frac{{\partial u_{y} }}{\partial x} + \frac{{\partial u_{x} }}{\partial y}} \right)^{2} } \right] + \left[ {\left( {\frac{{\partial u_{z} }}{\partial y} + \frac{{\partial u_{y} }}{\partial z}} \right)^{2} } \right] + \left[ {\left( {\frac{{\partial u_{x} }}{\partial z} + \frac{{\partial u_{z} }}{\partial x}} \right)^{2} } \right] \hfill \\ - \frac{2}{3}\left[ {\frac{{\partial u_{x} }}{\partial x} + \frac{{\partial u_{y} }}{\partial y} + \frac{{\partial u_{z} }}{\partial z}} \right]^{2} \hfill \\ \end{gathered} $$

where *x*, *y* and *z* are the coordinate axes.

#### 3S configuration

**Figure 1:**
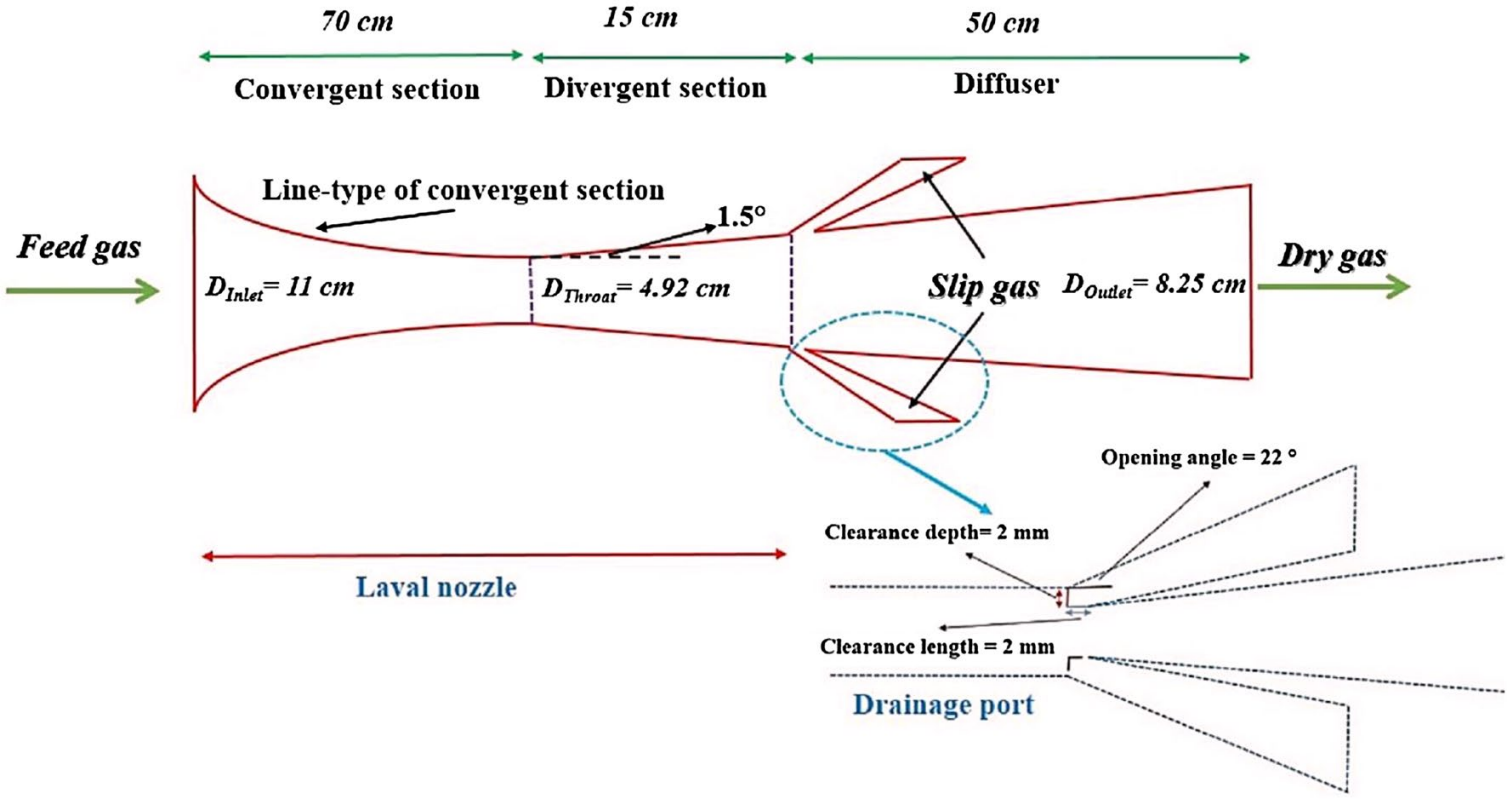
The schematic of employed 3S.

The 3S is composed of a Laval nozzle, a swirler, a drainage port and a diffuser. The Laval nozzle is constructed from three main sections: the convergent part (subsonic section), throat and the divergent section (supersonic section). The role of the converging section is accelerating the natural gas. Several structures can be considered for the convergent section of the nozzle^22^ such as Witoszynski curve, Quintic polynomial curve and Bi-cubic parametric curve. The line type of divergent part of the Laval nozzle was calculated by the following formula:

6 $$ L = \left( {r_{out} - r_{th} } \right)\tan \left( {{\raise0.7ex\hbox{$\varphi $} \!\mathord{\left/ {\vphantom {\varphi 2}}\right.\kern-0pt} \!\lower0.7ex\hbox{$2$}}} \right) $$

where *φ, r*_*out*_*, r*_*th*_ and* L* are the divergence angle, outlet radius, throat radius and divergence length of the nozzle, respectively. The structure of the optimal 3S is presented in Fig. 1. One of the crucial parameters which has an important influence on the system performance is configuration of the swirler. In the previous work published by the authors^6^, a new structure for generation of swirl flow was presented. In this structure, the swirl flow was generated using side injection (passive method). While, in the present work, a swirler is located at the inlet of the separator to generate the required centrifugal forces. The swirler contains some parallel vanes along with a central ellipsoid. The length of the optimal swirler is 12.5 cm, the number of static vanes is 6 and the vane angle is 45°. The static vanes profile curve were designed by Eq. (7)^13^:

7 $$ y = \sqrt {\left( {{\raise0.7ex\hbox{${0.5}$} \!\mathord{\left/ {\vphantom {{0.5} {\sin \left( {{\theta \mathord{\left/ {\vphantom {\theta 2}} \right. \kern-0pt} 2}} \right)}}}\right.\kern-0pt} \!\lower0.7ex\hbox{${\sin \left( {{\theta \mathord{\left/ {\vphantom {\theta 2}} \right. \kern-0pt} 2}} \right)}$}}} \right)^{2} - \left( {x - 0.5} \right)^{2} } - \frac{0.5}{{\tan \left( {{\theta \mathord{\left/ {\vphantom {\theta 2}} \right. \kern-0pt} 2}} \right)}} $$

where *θ* is the vane angle. The vane angle is defined as the angle between the outlet angle of the static vane and the axial direction. In this study the opening angle of the drainage port is 22°^58^, the divergent angle is 1.5° and the clearance length and depth of the drainage port is about 2 mm. After the structural optimization of the 3S using the CFD analysis, the optimized structure is used instead of the JT process. In this step, the investigated unit was optimized from a process point of view. Therefore, the aim of this article is the simultaneous use of structural modification and process optimization to improve the amount of NGL recovery with the minimum possible energy consumption. In this study, COMSOL Multiphysics software (version 5.4) and ASPEN PLUS (version 11) simulation software were used for optimizing investigated gas plant.

#### Mesh independency

The mesh quality plays an important role in the accuracy of the developed model. Grid independence test was conducted to ensure the accuracy of the simulation results. Five different mesh sizes including extremely coarse, coarser, coarse, normal and fine were investigated to achieve mesh independency. The tetrahedral mesh was used for the whole length. The grid independence test was conducted by Arina’s pressure data as a criterion. The results of the mesh independency test demonstrated that the normal mesh sizes provided the required accuracy for the studied 3S. Generally, the normal and fine grids practically lead to the identical solution. Consequently, normal grid size was selected for this numerical solution. The Grid Convergence Index (GCI) for the normal mesh size was also calculated by the Eq. (8)^59,60^:

8 $$ GCI_{21} = \frac{{F_{s} \left| \varepsilon \right|}}{{r_{21}^{p} - 1}} $$

It was observed that for all of the considered data on the Arina’s pressure profile the GCI for normal mesh size was as follows:

9 $$ \frac{{GCI_{32} }}{{r^{p} GCI_{21} }} \approx 1 $$

In conclusion, based on conducted analysis the grid independency was obtained for the normal mesh size. The developed equations were discretized by the finite element method, and the PARDISO solver was employed for the time-dependent equations. Furthermore, the convergence criterion was 10^–4^ for all of the developed equations. The pressure boundary conditions were considered for the inlet and outlet of the nozzle and about 60–80% of inlet pressure will be recovered at the diffuser. In addition, the adiabatic and no-slip boundary conditions were assigned for the separator’s wall.

#### Turbulence model

The natural gas flow through the 3S is turbulent and compressible. Furthermore, the swirl flow was produced by the static vanes at the inlet of the nozzle. The comparison of pressure profile between prediction of different turbulence models and Arina’s data showed that the V2-f turbulence model was more accurate than the κ-ε and κ-ω turbulence model in predicting Arina^’^s data. The calculated AARD% for κ-ε, κ-ω and V2-f turbulence models were equal to 25.39, 20.56 and 8.65%, respectively. The κ-ε turbulence model is not suitable for gas with high swirl flow^61^. Therefore, the compressible V2-f turbulence model was employed to analyze the swirl flow inside the 3S as follow:

10 $$ \rho \left( {u.\nabla } \right)k = \nabla .\left[ {\left( {\mu + \frac{{\mu_{T} }}{{\sigma_{K} }}} \right)\nabla k} \right] + P_{k} - \rho \varepsilon $$

11 $$ \rho \left( {u.\nabla } \right)\varepsilon = \nabla .\left[ {\left( {\mu + \frac{{\mu_{T} }}{{\sigma_{\varepsilon } }}} \right)\nabla \varepsilon } \right] + \frac{1}{\tau }\left( {C^{\prime}_{\varepsilon 1} \left( {\zeta ,\alpha } \right) - C^{\prime}_{\varepsilon 2} \left( {k,\varepsilon ,\alpha } \right)\rho \varepsilon } \right) $$

12 $$ \rho \left( {u.\nabla } \right)\zeta = \nabla .\left[ {\left( {\mu + \frac{{\mu_{T} }}{{\sigma_{\zeta } }}} \right)\nabla \zeta } \right] + \frac{2}{k}\left( {\alpha^{3} \mu + \frac{{\mu_{T} }}{{\sigma_{k} }}} \right)\nabla k.\nabla \zeta + \left( {1 - \alpha^{3} } \right)f_{w} + \alpha^{3} f_{h} - \frac{\zeta }{k}P_{k} $$

13 $$ \mu_{T} = \rho C_{\mu } k\zeta \tau , \, \tau = \max \left[ {\frac{k}{\varepsilon },C_{\tau } \sqrt {\frac{v}{\varepsilon }} } \right] $$

14 $$ P_{k} = \mu_{T} \left[ {\nabla u:\left( {\nabla u + \left( {\nabla u} \right)^{T} } \right) - \frac{2}{3}\left( {\nabla .u} \right)^{2} } \right] - \frac{2}{3}\rho k\nabla .u $$

where *k, µ*_*T,*_* ε, α, P*_*k*_, *G* and *ζ* show the turbulent kinetic energy, turbulent viscosity, turbulence dissipation rate, elliptic blending function, production term, Reciprocal wall distance and turbulent relative fluctuations, respectively. The *C*_μ_, *C*_τ_, $C^{\prime}_{\varepsilon 1}$, $C^{\prime}_{\varepsilon 2}$, *σ*_k_, *σ*_ε_ and *σ*_ζ_ are the constant in the above equations. The reader is referred to the COMSOL Multiphysics help for more detail about the V2-f turbulence model.

#### Particle tracing model

After the expansion through the converging part of Laval nozzle, the two-phase fluid passes through the throat. Under the influence of induced centrifugal force, the liquid droplets are thrown toward the separator’s wall and form a liquid film. Due to the strong centrifugal force, even submicron water and natural gas condensate droplets will be pushed to the separator wall^62^. To evaluate the separation performance of the investigated 3S, a sufficient number of spherical droplets were injected at the inlet of the 3S. The particle tracing model was used to track the droplets behavior in the 3S. The behavior of the liquid droplets in the 3S can be described by the force analysis. The inserted forces in a liquid droplet were considered by the Eq. (15):

15 $$ F = \frac{{d\left( {mv} \right)}}{dt} $$

where *m *and *v* are the mass and volume of liquid droplets, respectively. The drag force (*F*_*D*_) was determined using the Schiller-Neuman equation^63^. In this study, two specified droplet sizes (2 µm and 4 µm^64^) were employed to analyze the phase separation inside the 3S. The uniform and spherical liquid droplets were considered in this study. In addition, in a 3S, the role of centrifugal force is more significant compared to the gravity force^65^. Therefore, the gravity force was neglected in this simulation.

Part of the liquid droplets will be separated at the collection point and remaining droplets will be entrained by the gas flow toward the nozzle outlet. The determination of separation efficiency is the most common method of analyzing the performance of the supersonic separation technology. The collection efficiency (*N*) of this equipment can be described as the number of the separated droplets divided by the total number of the injected droplets at the inlet of the 3S as follow:

16 $$ N = 1 - Entrainment = \frac{{n_{separated} }}{{n_{total} }} \times 100\% $$

In addition, to clarify the condensation efficiency of 3S for each component, a parameter which is called component collection efficiency (*η*) was defined as follow:

17 $$ \eta = \frac{{x_{i - in} - x_{i - out} }}{{x_{i - in} }} \times 100\% $$

where *x*_*i-in*_ is the mole fraction of component i at the inlet and *x*_*i-out*_ is the mole fraction of component i at the outlet. Furthermore, the pressure loss ratio (PLR) (γ) through the 3S was defined as follow:

18 $$ \gamma = \frac{{P_{in} - P_{out} }}{{P_{in} }} \times 100\% $$

where *P*_*in*_ and *P*_*out*_ are the inlet pressure and outlet pressure, respectively. Furthermore, the cooling performance of each investigated structure was defined by Eq. (19):

19 $$ C = \frac{{T_{inlet} - T_{\min } }}{{T_{inlet} - T_{\min - overall} }} \times 100\% $$

where *C, T*_*inlet*_, *T*_*min*_, and *T*_*min-overall*_ are the cooling performance, inlet temperature, the minimum temperature inside the 3S equipped with swirler, and the minimum temperature inside the simple optimized 3S (without swirler), respectively. In order to consider the simultaneous effect of both cooling performance (*C*) and collection efficiency (*N*), the separation efficiency parameter (*S*) was defined as follows:

20 $$ S = N \times C $$

### Thermodynamic analysis

Natural gas after extraction from underground with the operating temperature of 30 °C, pressure of 121 bar and gas flow rate of 4 MMSCM enters into the investigated NGL recovery unit. The under-studied gas plant operated based on the dehydration by mono-ethylene glycol (MEG) and NGL recovery by the three-stage throttling process. The first stage JT valve and low temperature separator (LTS) (V-200) is not the concern of this paper, while the exergy efficiency and NGL recovery rate of second stage and third-stage JT valve and LTS are investigated comprehensively. One of the most important parts of the gas plant is the low temperature generation for NGL recovery. Therefore, starting from the throttling process, three new schemes based on the 3S process were suggested for the NGL recovery. In the proposed method for the cooling, liquefaction and phase separation, the compact 3S equipment was used. Furthermore, the hydrate inhibitor and its regeneration system were eliminated from the studied gas plant due to the very low residence time of gas flow through the 3S. The working principle of both processes are based on the self-refrigeration. This cold energy for natural gas liquefaction comes from the pressure energy of the feed stream.

The Joule–Thomson coefficient (JTC) (*µ*_*JT*_) is defined as the rate of temperature variation to pressure variation in a throttling process. This parameter is defined as follow:

21 $$ \mu_{JT} = \left( {\frac{\partial T}{{\partial P}}} \right)_{H} = \frac{V}{{C_{P} }}\left( {\alpha T - 1} \right) $$

where *V* is the molar volume, *α* is the thermal expansion coefficient, *C*_*P*_ is the specific heat capacity and *T* is the temperature. Furthermore, the cooling depth was defined as follow:

22 $$ Cooling \, depth = \frac{{T_{Inlet} - T_{\min } }}{{P_{Inlet} - P_{Outlet} }} $$

where *P*_*Inlet*_ and *T*_*Inlet*_ are the operating pressure and temperature before expansion and *P*_*Outlet*_ and *T*_*min*_ are the operating pressure after expansion and minimum achievable temperature through the system, respectively.

There are various modifications which can be employed to improve the exergy efficiency and NGL recovery rate. These modifications are as follow:

1. Optimizing of operational condition
2. Modifying the 3S configuration including structure of swirler and nozzle line-type.
3. Modifying the process scheme.

Therefore, the NGL recovery rate and exergy efficiency has been selected as the objective functions, and the intermediate pressure ratio, internal configuration of the 3S and gas plant scheme were defined as the variables. Consequently, the sensitivity analysis was conducted to find the influence of each variable on the objective functions. In order to optimize the operational condition several scenarios for intermediate pressure ratio were also defined (Table 1).

**Table 1:** Scenarios examined for intermediate pressure ratio.

Scenarios	P1 (Bar)	P2 (Bar)	P3 (Bar)	ΔP1 = P1–P2	ΔP1 = P2–P3	ΔP1/ΔP2
Scenario 1	121	110	69.4	11 bar	40.6 bar	0.27
Scenario 2	121	100	69.4	21 bar	30.6 bar	0.68
Scenario 3	121	90	69.4	31 bar	20.6 bar	1.50
Scenario 4	121	80	69.4	41 bar	10.6 bar	3.86

#### Process simulation

**Figure 2:**
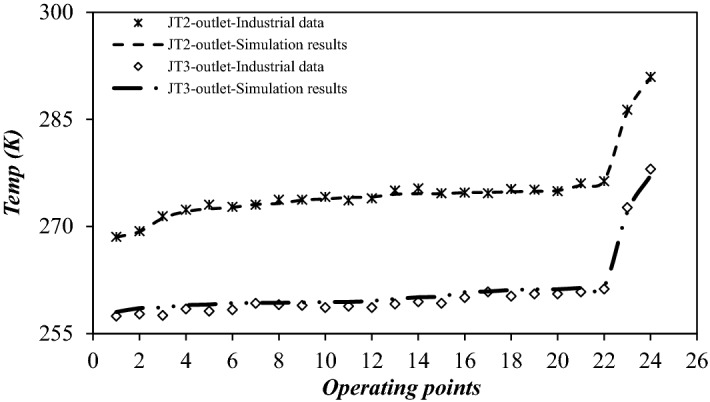
Comparison between simulation result and gas plant data for various operating condition.

**Figure 3:**
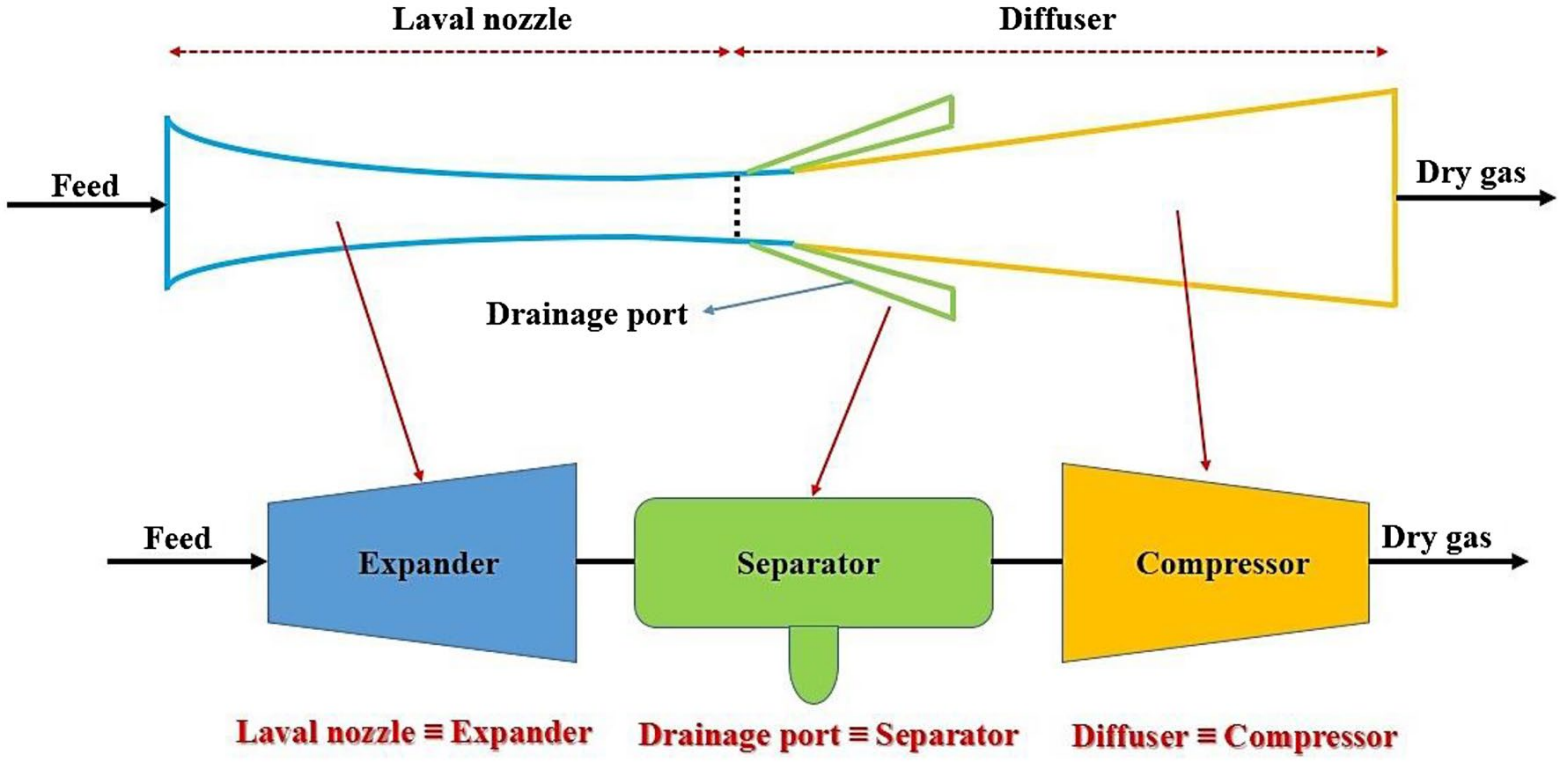
Process equipment equivalent to a 3S.

In this study, the operating condition and plant configuration were optimized by ASPEN PLUS simulation software for a fixed inlet and outlet boundary condition. The output of employed simulation software was compared with the recorded industrial data in this plant. It was observed that there is a good agreement between simulation results and industrial data (Fig. 2). As the 3S module was not available in the ASPEN PLUS, it was modeled by COMSOL Multiphysics software, then, the results of CFD analysis were imported into the ASPEN PLUS software to consider the 3S process. The role of 3S was defined using three process equipment including an expander, a separator and a compressor^66^ (Fig. 3). It is obvious, the phase change and liquid formation in the 3S happens in the Laval nozzle. The reason for this phase change and the formation of the liquid phase is the very low temperature that occurs due to the supersonic expansion in the Laval nozzle. In order to consider the role of the Laval nozzle in the ASPEN PLUS simulation software, the expander process equipment was considered (Fig. 3). Therefore, using the SRK equation of state (EoS) and the expander process module, the role of the Laval nozzle was completely simulated in the ASPEN PLUS simulation software. Then the liquid phase formed due to the expansion is separated in the multiphase separator. Therefore, the whole gas plant was simulated by ASPEN PLUS simulation software, incorporating the 3S function, imported from COMSOL Multiphysics software.

#### Selection of suitable equation of state

An appropriate EoS should be considered to determine the natural gas thermo-physical properties in supersonic flow. The Soave–Redlich–Kwong (SRK) EoS was used in this study due to its high accuracy. The accuracy of various EoSs including three-parameter EoSs—Esmaeilzadeh and Roshanfekr, Harmen and Knapp, and Patel and Teja—and two-parameter equations—Nasrifar and Moshfeghian, Peng-Robinson (PR), and SRK was thoroughly investigated elsewhere^67^. Based on the obtained results, SRK EoS showed the most accurate prediction of the JTC and the inversion curve of natural gas components. Therefore, the SRK EoS was used for description of natural gas behavior^68,69^.

#### Exergy efficiency

In this work, the exergy analysis was employed to investigate the efficiency of various process equipment such as heat exchanger (HX), separator and JT valve. Exergy analysis was developed by the second law of thermodynamics and can be employed to find the system irreversibility. By determining the exergy of each stream, the exergy efficiency and exergy destruction of each process equipment can be calculated. Using the calculated enthalpy and entropy of each stream the exergy of each stream was determined by Eq. (23):

23 $$ Ex = (H - H_{Ref.} ) - T_{Ref.} (S - S_{Ref.} ) $$

In addition, the exergy destruction can be calculated by Eq. (24):

24 $$ Ex_{d}  = \sum {\left( {1 - \frac{{T_{{Ref}} }}{{T_{i} }}} \right)} Q - W + \sum {\mathop m\limits^{ \cdot } _{{Inlet}} Ex_{{Inlet}} }  - \sum {\mathop m\limits^{ \cdot } _{{Outlet}} Ex_{{Outlet}} }   $$

where *H* is the enthalpy, *S* is the entropy, the *S*_*Ref*_ and *H*_*Ref*_ are the reference entropy and enthalpy, *T*_*i*_ is the specified temperature where heat transfer occurred. *Q* is the rate of heat transfer, $\mathop m\limits^{ \cdot }$ is the mass flow rate and *W* is the conducted work. The total exergy destruction can be obtained by the Eq. (25):

25 $$ Ex_{d} = Ex_{1} + Ex_{2} + Ex_{3} + Ex_{4} $$

A reference temperature equal to 293.15 K was assumed in this paper. The efficiency of 3S and JT valve were determined by Eq. (26)^70^:

26 $$ \varepsilon = {\raise0.7ex\hbox{${e_{Out} }$} \!\mathord{\left/ {\vphantom {{e_{Out} } {e_{In} }}}\right.\kern-0pt} \!\lower0.7ex\hbox{${e_{In} }$}} $$

where *e*_*Out*_ and *e*_*In*_ are the exergy after and before expansion process, respectively.

#### Process configuration

**Figure 4:**
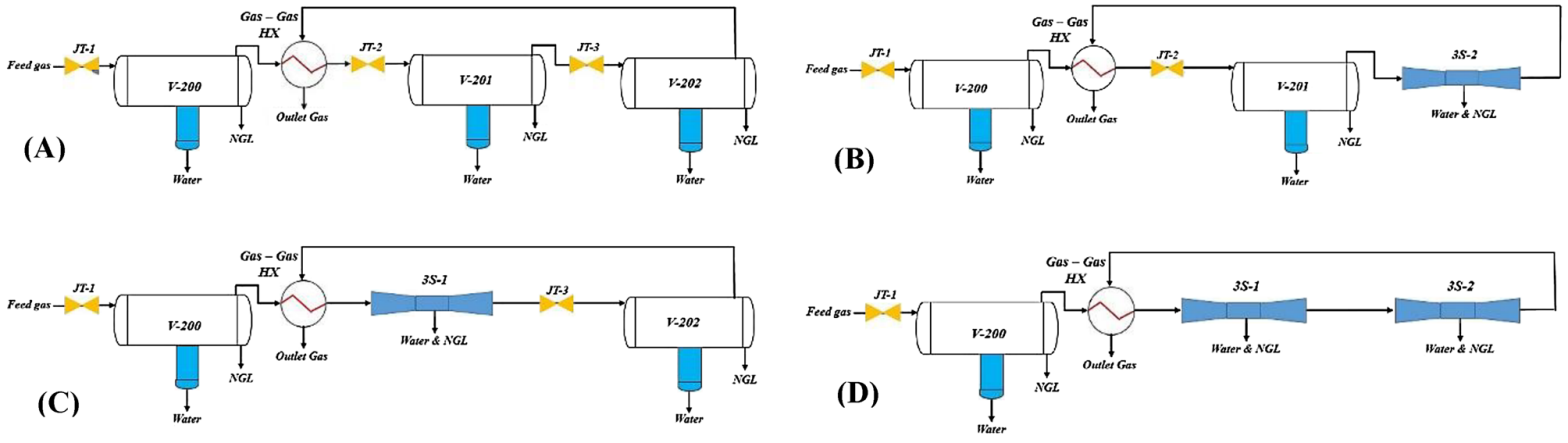
Schematic diagram of (**A**) JT process (configuration A), (**B**) integrated JT + 3S process (configuration B), (**C**) integrated 3S + JT process (configuration C), and (**D**) 3S process (configuration D).

In this study, four different configurations based on the JT process, 3S process and their combination were defined to select the optimal configuration. For the original configuration (configuration A, Fig. 4), the JT valves were employed to decrease the natural gas temperature to partially condense the NGL entering the LTS. In the LTS, the liquid phase is separated, while the gas phase is directed toward the next stage refrigeration process. A schematic diagram of the investigated gas plant is presented in Fig. 4A. It should be emphasized that there are three JT valves in this plant and one of them (JT-1) may be out of service. Consequently, the liquefaction is performed in two stages. For all of the considered configurations presented in Fig. 4, the separation process initiates with gas cooling using the shell and tube HX. The outlet stream from the tube side of HX (HX-200), passes through the second stage JT valve (JT-2), where partial condensation with liquid phase separation occurs at the outlet of this valve. After that, the outlet of the second stage JT valve was expanded further in the third stage valve (JT-3) to separate lighter HCs in V-202. The outlet dry gas from V-202 passed through the shell side of HX and its temperature is increased. Glycols are suitable inhibitors due to the presence of the hydroxyl group. The MEG was injected before the JT-2 and JT-3. The free water in the natural gas stream will be separated by MEG to prevent hydrate formation after the JT valves.

Four process configurations can be defined for investigated gas plant: 1- JT + JT processes (Fig. 4A), 2- JT + 3S processes (Fig. 4B), 3- 3S + JT processes (Fig. 4C), and 4- 3S + 3S process (Fig. 4D). Although, modifying process scheme is an expensive method for improving the system performance, but this is a common method in these processes. Therefore, in this section, three new layouts (Configurations B, C and D) for NGL recovery were introduced and the advantages and disadvantages of each case were evaluated. In these configurations JT valve and LTS were replaced by a 3S, to improve the system performance.

## Result and discussion

**Figure 5:**
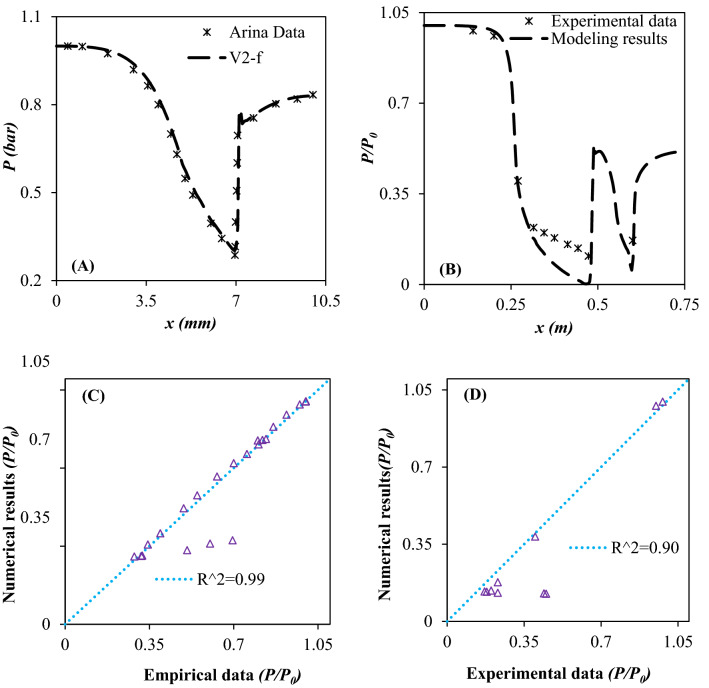
(**A, B**) Comparison between CFD modeling prediction and empirical data^71,73^, (**C, D**) Error analysis using root-mean-square (*R*^*2*^) method.

The goal of this study is to maximize the NGL recovery rate and minimize the energy consumption by improving the system performance. Optimization of operating conditions and design parameters is one of the most familiar methods of improving the system performance. Therefore, a novel separation process was proposed to be replaced by the existing JT valve and LTS. Then, the influence of operating parameters on the efficiency of original process scheme (configuration A) and other modified process schemes (configuration B, C and D) was analyzed and discussed. Before using any developed model, its accuracy should be evaluated. Consequently, it is crucial to validate the developed CFD model. Figure 5 shows how the model output is generally consistent with Arina’s data^71^ with AARD% of 8.65%. In addition, the root mean square (*R*^*2*^)^72^ was employed to further analyze the accuracy of the developed CFD model. The root mean square (*R*^*2*^) showed (Eq. (27))^72^ the errors between CFD output and empirical results. The predicted pressures by CFD modeling agree well with the Arina’s data^71^ with *R*^*2*^ of approximately 0.99.

27 $$ R^{2} = 1 - \frac{{\sum\nolimits_{i = 1}^{n} {\left( {a_{i} - h_{i} } \right)^{2} } }}{{\sum\nolimits_{i = 1}^{n} {\left( {h_{i} } \right)^{2} } }} $$

where *h*_*i*_ is the CFD result, n is the number of investigated data and *a*_*i*_ is the experimental data. Therefore, the developed model is precise. For Arina’s nozzle, *M*_inflow_ = 0.239543, and *P*_*exit*_ = 0.83049*P*_*inflow*_, the gas flow in the convergent section is subsonic, enhances up to sonic velocity in the throat and reaches supersonic velocity in the divergent section. In this nozzle, the gas expanded from the supercritical phase to the gas phase without formation of liquid phase^71^. In addition, in order to analyze the ability of the presented model in describing the phase change process, another series of empirical data^73^ was also used. Eriqitai et al.^73^ employed a gas compressor to generate high pressure air. Furthermore, an atomizer was used to generate micro-water droplets and imported in the air flow. These micro-water droplets were liquefied at the Laval nozzle. In order to further verify the developed model, experimental data of Eriqitai et al.^73^, which are accompanied by phase change, were used. It can be observed that the developed CFD model predicted suitably the trend of pressure distribution with *R*^*2*^ of approximately 0.90.

### Optimization of 3S configuration using the CFD modeling

The main goal of this section was to optimize the effective parameter on the separation efficiency of investigated 3S using the CFD modeling. Consequently, the structure of nozzle and swirler should be optimized to improve the cooling performance, enhance the collection efficiency and reduce the energy loss through the 3S. Therefore, firstly the cooling performance and then the collection efficiency were improved.

#### Optimization of nozzle structure

The main role of Laval nozzle is providing the suitable temperature and pressure for the liquefaction of condensable components like water vapor and heavy HCs. The Laval nozzle with optimal cooling performance is capable of condensing more HCs compared to the nozzle with lower cooling performance. The line-type of the convergent and divergent section of the Laval nozzle influences the flow stability and cooling performance. To optimize the structure of the convergent section, the profile of the convergent section was designed by the Witoszynski curve, Quintic polynomial curve, linear curve and Bi-cubic curve^22^, respectively. The simulation results illustrated that the Witoszynski curve showed the best cooling performance (lowest average cooling temperature) compared to others. Many researchers also found that the optimal curve for the convergent section of the Laval nozzle is the Witoszynski curve^74–76^. Furthermore, both linear and curved wall formulas (19) were employed for design of the diffuser section of the nozzle. The curved wall diffuser showed better cooling performance and uniformity in velocity profile than linear wall diffuser. Therefore, the Witoszynski curve and the curved wall formula were selected for designing the convergent section and diffuser section of the 3S, respectively. For an optimal nozzle, the effect of different convergent length (40, 50, 60 and 70 cm) and diffuser length (30, 40 and 50 cm) on the cooling performance was also investigated numerically. Simulation results showed that, increasing both the convergent length and diffuser length improved the cooling performance. For the optimized 3S, the lowest achievable temperature was about 197.55 K, which provided appropriate conditions for liquefaction of condensable components like water vapor and heavy HCs.

#### Optimization of swirler structure

Swirl intensity and induced centrifugal force play a crucial role in the separation of liquid droplets from the gas phase. In this section, the influence of installing swirler on the separation performance of the 3S was investigated. The optimization of swirler structure is a crucial factor in improving system performance. Several geometrical parameters of the swirler including swirl angle, number of static vanes and swirler length were varied and the optimal value for each case was selected.

**Figure 6:**
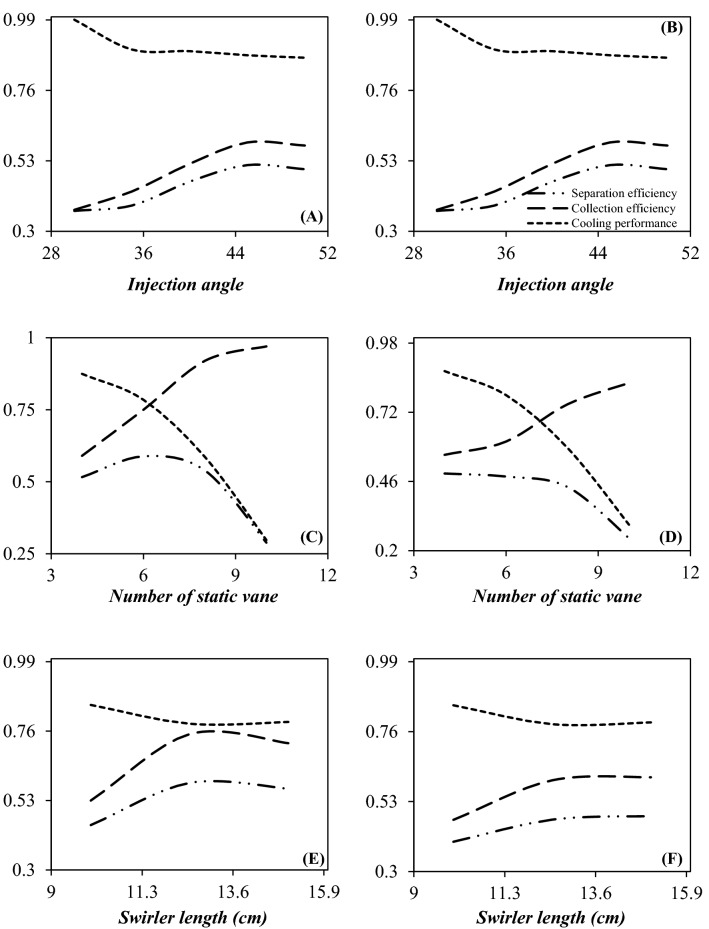
The influence of injection angle (**A, B**), number of static vane (**C, D**) and swirler length (**E, F**) on the cooling performance, collection efficiency and separation efficiency of water (**A, C** and **E**) and HC (**B, D** and **F**) droplets with the diameter of 2 µm.

The swirl angle is an important factor for generation of centrifugal force. The effect of various swirl angles on the flow behavior and separation efficiency was investigated numerically considering angles of 30°, 35°, 40°, 45° and 50°, respectively. Figure 6A and B depict the collection efficiency, cooling performance and the separation efficiency as a function of the injection angle. It can be observed that, increasing the swirl angle has a positive influence on the collection efficiency and negative influence on the cooling performance. Therefore, the existence of the swirl velocity through the nozzle destroyed the expansion characteristic of the 3S. This means that it is crucial to balance the collection efficiency and cooling performance to obtain a higher separation efficiency by the 3S. Figure 6A and B showed that there is an optimal swirl angle to maximize the separation efficiency of the investigated 3S. Therefore, the swirl angle of 45° was selected as the optimum value and the behavior of natural gas through the 3S was predicted for this swirl angle (θ = 45°).

**Figure 7:**
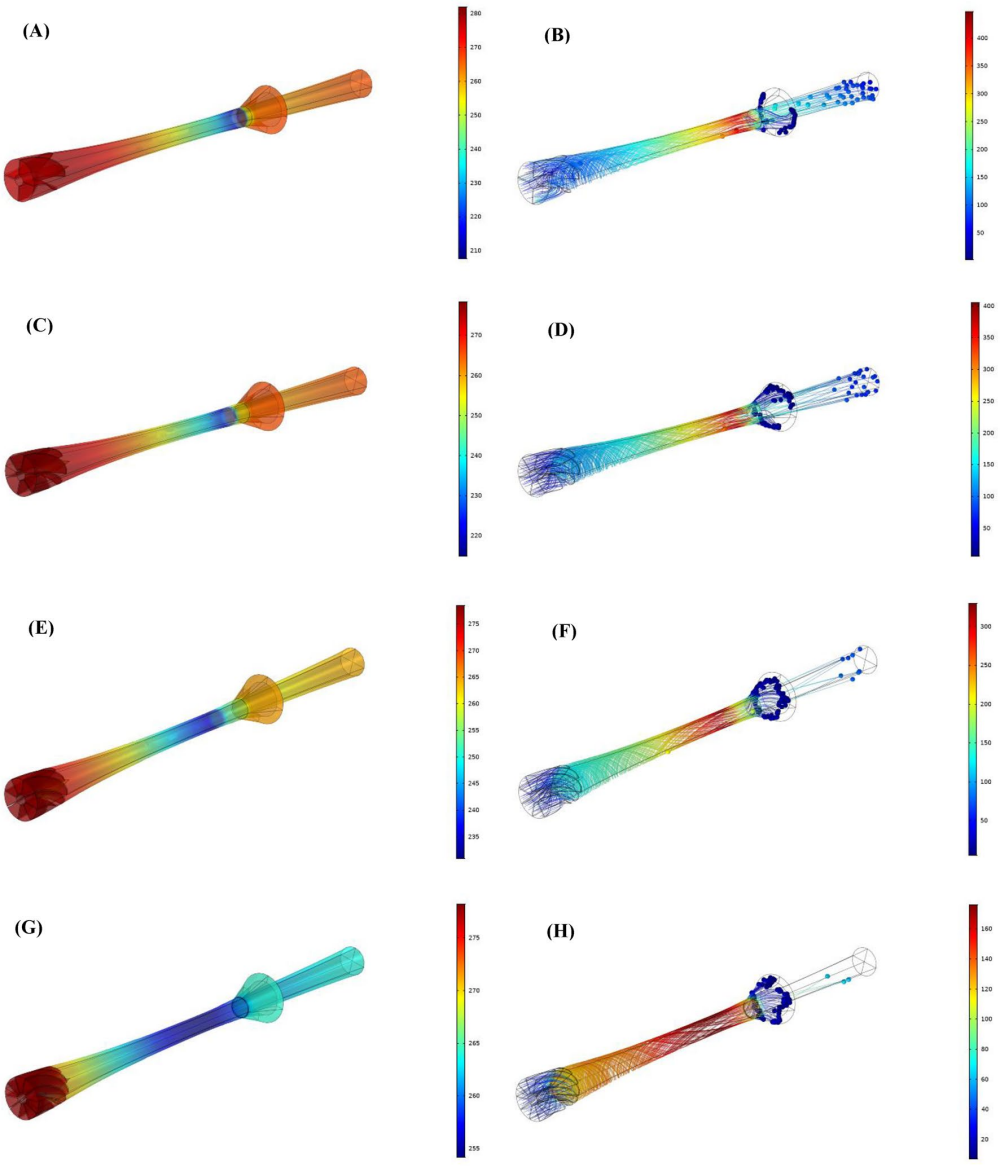
The influence of number of static vanes on the (**A, C, E** and **G**) temperature profile (K) and (**B, D, F** and **H**) liquid droplet behavior (dp = 2 µm).

Figure 6C and D show the influence of the number of static vanes on the cooling performance, collection efficiency and separation efficiency of water and HCs droplets when the number of static vanes are 4, 6, 8 and 10. It can be seen that (Fig. 7), for both water and HCs droplets with the increase of the vane number, the collection efficiency increased. Contrary to this, increasing the number of static vanes deteriorated the cooling performance and increased the energy loss through the system. Furthermore, simulation results showed that if the number of static vanes enhanced more than a specified value, it disturbed the gas flow and the separation efficiency deteriorated. Consequently, it is necessary to balance the collection efficiency and cooling performance during the design of the swirler. In addition, as shown in Fig. 6C and D for HC droplets, four and six static vanes provided relatively the same efficiency. However, for water droplets, six static vanes showed the highest separation efficiency. Therefore, six static vanes were employed to balance the swirl intensity and cooling performance through the 3S. Consequently, the optimized swirler contained 6 static vanes by swirling angles of 45°. Furthermore, to investigate the influence of swirler length on the separation performance, the 3S was simulated for this case (Fig. 6E and F). It can be observed that the highest separation efficiency was obtained at the length of 12.5 cm. Therefore, the parameters of the considered swirler are: swirl angle = 45°, number of static vanes = 6 and swirler length = 12.5 cm, respectively.

The effect of inserting an inner body on the expansion properties and separation efficiency was also investigated in this section. Based on the conservation law of angular momentum, due to the reduction of radius in the divergent section of the nozzle, the tangential velocity is enhanced. Therefore, inserting an inner body in the divergent part of the nozzle improved the separation efficiency by enhancing the centrifugal force^13^. Simulation results showed that installing the internal body slightly improved the separation efficiency. Consequently, the optimized 3S showed appropriate separation efficiency with the collection efficiency of 77% and 60% for water and HC droplets of 2 µm diameter.

#### Optimization of plant configuration and operating condition

In this section, several plans have been proposed for optimization of investigated gas plant by modification of operating parameters and plant scheme. The plant scheme was modified by employing 3S, whose structure was optimized in the previous section, instead of JT valve and LTS (configuration B, C and D). In order to investigate the effect of operational parameters on the defined objective functions (NGL recovery rate and exergy efficiency), four scenarios for intermediate pressure ratio were also defined (Table 1).

#### NGL recovery rate and the cooling depth

**Figure 8:**
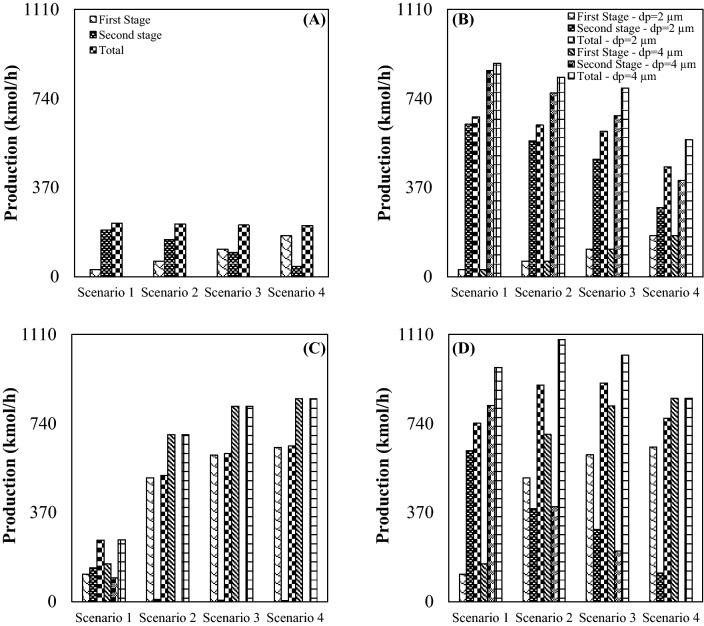
NGL recovery rate for various scenarios, droplet diameter and configurations: (**A**) configuration (**A, B**) configuration (**B, C**) configuration (**C** and **D**) configuration (**D**).

The aim of this section is modification of configuration of investigated gas plant to produce more NGL compared to the conventional JT process (configuration A). It is evident that the maximization of NGL recovery rate resulted in the improvement of the plant efficiency. Therefore, the 3Ss were installed instead of the JT valves and LTSs to enhance the NGL recovery rate. Figure 8 presents the amount of NGL recovery rate for different process schemes. The simulation results demonstrated that all of the proposed configuration (configuration B, C and D) improved the NGL recovery, better than existing configuration (configuration A). Based on the simulation results, at the optimal operating condition and for droplet diameter of 2 µm, the NGL recovery rate of configuration A, B, C and D reached 222 kmol/h, 662.8 kmol/h, 646.9 kmol/h and 908.5 kmol/h, respectively. Consequently, configuration D provided a higher NGL recovery rate at the same operating condition. At the optimal condition, the NGL recovery rate of the configuration D (for d_p_ = 2 µm) was improved by 309.2% compared to the original design (Configuration A). Furthermore, based on Fig. 8, lighter HCs concentration is higher for recovered NGL by 3S process (configuration D) than the JT process (configuration A). Consequently, the configuration D is the most efficient process scheme for natural gas liquefaction. Another option is employing integrated configuration (configuration B and C) instead of existing configuration (configuration A). Integrated configuration provided the condition that light and heavy HCs can be recovered by the 3S and JT process, respectively. Generally, the separated NGL from the 3S contained light and heavy HCs. However, the separated NGL from the JT process contained only the heavy HCs. Furthermore, it can be observed that the NGL recovery is enhanced for integrated configuration compared to the JT process. At the optimal condition (for d_p_ = 2 µm), the configuration B and C provided 198.5% and 191.3% higher NGL recovery rate than the configuration A. This improvement in the NGL recovery rate can be explained based on the minimum achievable temperature through the system.

The NGL recovery rate is a strong function of the minimum achievable temperature through the system. The main deficiency of the JT process is that it cannot decrease the operating temperature as low as the 3S process. In the original design (configuration A), a significant amount of energy should be consumed to achieve lower temperature through the system. However, by replacing the JT process by the 3S with similar energy consumption, lower temperature can be achieved. For example, when the JT valve was used for natural gas liquefaction, the lowest temperature was about 261.5 K (configuration A). However, when the 3S was used at the same operational condition the lowest temperature was about 196.9 K (configuration D). In addition, it can be said that with decreasing the minimum temperature, lighter components can be recovered.

Simulation results showed that the minimum temperature inside the 3S significantly decreased as the outlet pressure decreased. Therefore, in this plant, the intermediate pressure ratio plays an important role in improving the process efficiency. The performance of the JT process and supersonic separation process were investigated to compare their efficiencies. In addition, the integrated process (JT + SS) showed higher efficiency compared to the JT process. To investigate the effect of operational parameters on the system performance, the influence of different values of intermediate pressure ratio (Table 1) on the NGL recovery rate was also investigated. The increase of the pressure gradient through the 3S resulted in more temperature drop in the Laval nozzle. In this condition lighter HCs were liquefied that provided a higher NGL recovery rate. Although Fig. 8 showed that the 3S provided a higher NGL recovery rate if the PLR increased. Since the total pressure drops across the system is constant (ΔP_1_ + ΔP_2_ = constant), increasing the PLR in one stage leads to decreasing PLR in another stage. Therefore, the optimal scenario for intermediate pressure ratio for each configuration should be found. The simulation results indicated that the maximum NGL recovery rate was obtained for configuration A, B, C and D (d_p_ = 2 µm) at an intermediate pressure ratio of 0.27, 0.27, 3.86 and 1.5, respectively. Simulation results demonstrated that for configuration D, the NGL recovery rate was enhanced up to 22.4% by increasing the intermediate pressure ratio from 0.27 to 1.5. In addition, Fig. 8 shows for configuration D with the increase of the intermediate pressure ratio, the NGL recovery firstly increased and then decreased. Therefore, through design modification or process optimization, the performance of an NGL recovery unit can be improved. In addition, based on the sensitivity analysis, modifying the original configuration is the most efficient way of improving the NGL recovery rate.

**Figure 9:**
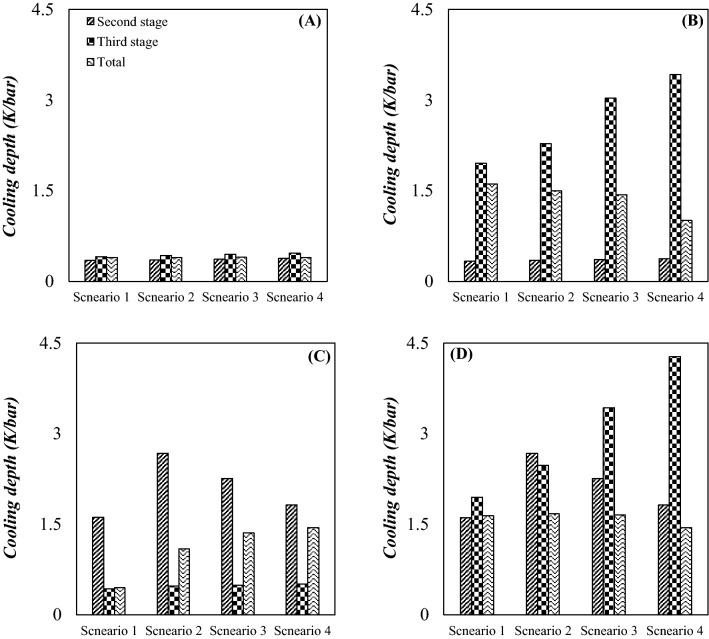
Cooling depth for different scenarios, and configurations: (**A**) configuration (**A, B**) configuration (**B, C**) configuration (**C** and **D**) configuration (**D**).

The natural gas capacity to hold liquid phase depends on its temperature and pressure. The cooling depth of the JT and 3S process is a function of the pressure gradient through these equipment. In addition, because the expansion through the 3S is close to the isentropic condition, the cooling depth using this process is higher than the JT process. The minimum temperature in the investigated JT process (configuration A) was about 261.5 K, which is not suitable for liquefaction of light HCs. Based on Fig. 9, the 3S process (configuration D) and integrated process (configuration B and C) provided higher cooling depth than JT process (configuration A). In addition, for configuration D higher NGL recovery rate was obtained. It can be observed that for the proposed configurations (configuration B, C and D), the deeper the cooling depth, the higher the NGL recovery rate. Based on this, scenario 1 in configuration B, scenario 4 in configuration C and scenario 3 in configuration D have the deepest cooling and greater NGL recovery rate. Furthermore, it can be seen that in configuration A and D, for identical pressure drops through the second stage and third stage, the temperature drop is higher at the third stage. This means that the JT effect was stronger in the third stage compared to the second stage. Consequently, as the methane content increases, the outlet temperature from JT process or 3S process decreases further. A similar result has been reported in previous published work^69^. Therefore, while at atmospheric pressure, adding a heavy component to a light component improved the cooling properties, but at the higher pressures this behavior may be different.

### Characterization of separation performance based on the phase envelop diagram, dew point depression and component collection efficiency

When the operating temperature becomes lower than the liquefaction temperature (dew point line), the natural gas starts to liquefy. Therefore, an accurate prediction of natural gas phase behavior is essential to design the separation process efficiently. The natural gas phase behavior can be characterized by the PED to find the dew point depression, the HCDP at specified operating conditions and the NGL recovery rate. The minimum achievable temperature through the 3S is an important factor which can influence the shape of PED. Based on the operating temperature and pressure and entrainment effect the produced natural gas can be single phase or two phase.

**Figure 10:**
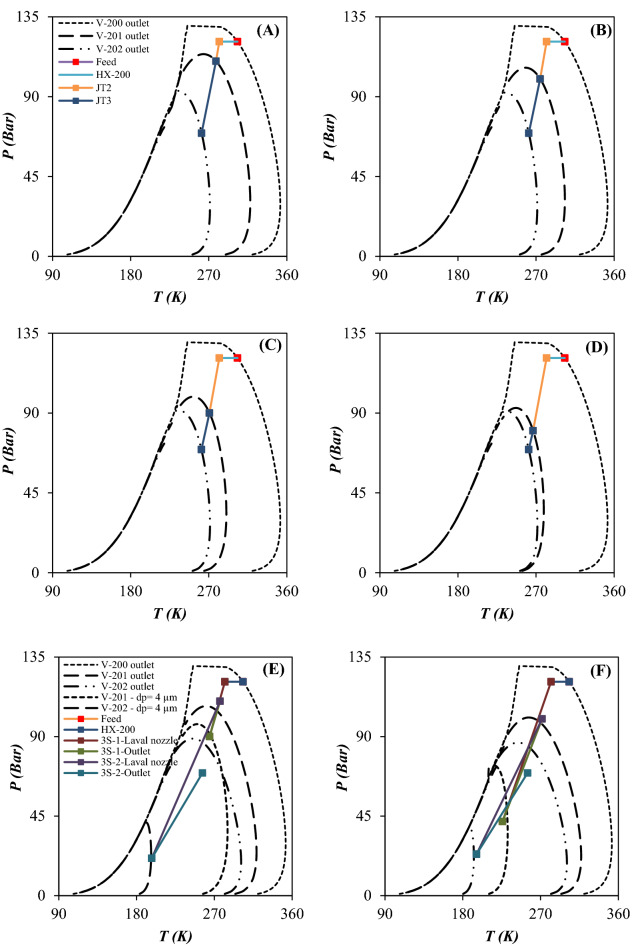

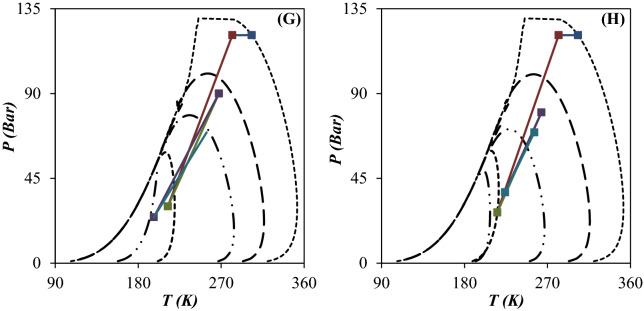
PED for configuration A (**A–D**) and D (**E–H**): (**A, E**) Scenario 1, (**B, F**) Scenario 2, (**C, G**) Scenario 3 and (**D, H**) Scenario 4.

**Figure 11:**
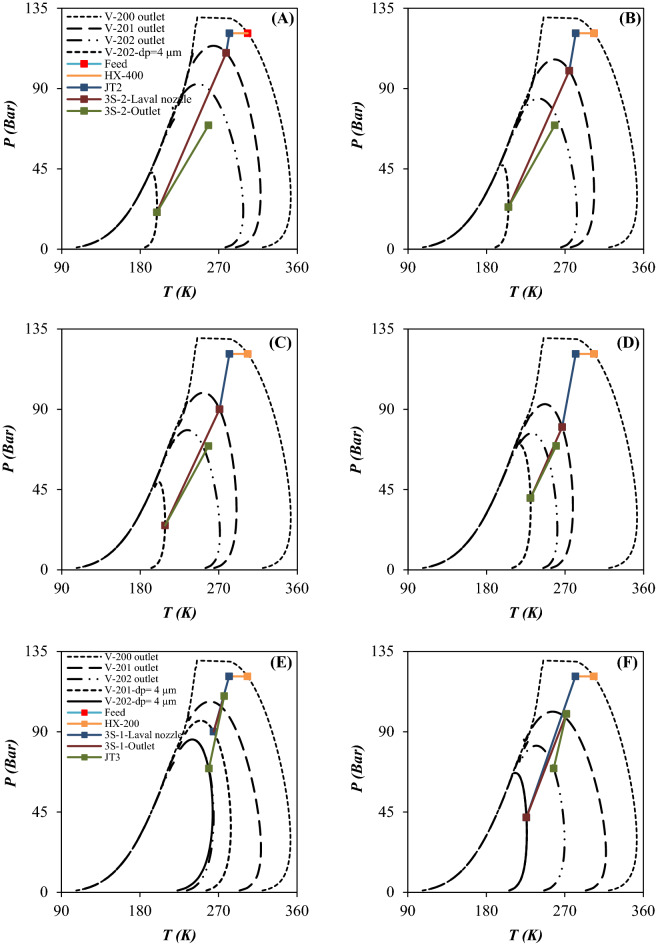

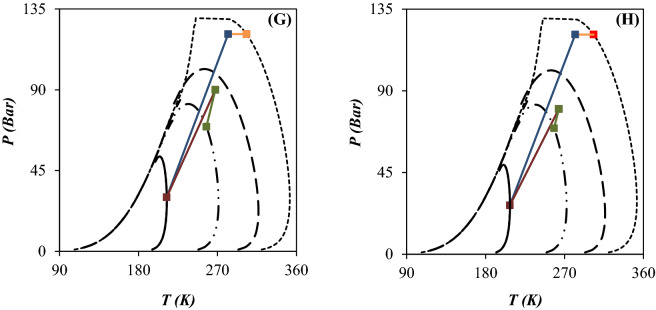
PED for configuration B (**A–D**) and C (**E–H**): (**A, E**) Scenario 1, (**B, F**) Scenario 2, (**C, G**) Scenario 3 and (**D, H**) Scenario 4.

In this study, the performance of the JT process and supersonic separation process were compared and the PED were plotted for each case (Figs. 10 and 11). Then, the influence of operating conditions on the PED of each case was discussed. To better compare the influence of employing the 3S instead of the JT process, the variation of PED is presented in Fig. 10. For the feed gas, the PED is very wide with a large domain which can be attributed to the existence of heavy HCs. These heavy HCs can be recovered easily by the temperature drop. Figure 10 compares the process line of JT process (configuration A) and 3S process (configuration D) and the location of inlet and outlet condition compared to the PED. The scope of the process line inside the PED determines the composition of heavier components of natural gas at the separator outlet and its dew point curve. According to the PED for the JT process, the pressure drop should be high enough to process line enter into the two-phase region. The analysis of the PED illustrated that the JT process could not produce enough gas condensate for a specified operating condition. In another words, when the NGL recovery was conducted using the JT processes, the process line entered into the PED slightly and the NGL recovery rate was negligible compared to the 3S process. Furthermore, the PED of outlet gas from the throttling process was wider than the 3S process. Consequently, the dew point of dry gas outlet from the 3S was lower than the throttling process. Based on this diagram, the separation of light HCs like ethane and propane by the JT process is not possible. Therefore, lower minimum temperature is required for liquefaction of these components. This issue can be explained based on the PED of considered natural gas streams. The easier way of comparing the performance of the JT process and the 3S process was to look at the location of the dew point line on the PED. As shown in Fig. 10, for an equal pressure gradient, the dew point line for the 3S moved to the left more than the JT process. Because the expansion process in the Laval nozzle is near to the isentropic process, the minimum achieved temperature by 3S process was lower than the isenthalpic processes like JT process. In addition, in the 3S process, the size of the liquid droplets has a significant effect on the position of the dew point curve. As can be seen, for droplets with a diameter of 4 μm, the PED moved much more to the left than for droplets with a diameter of 2 μm. Therefore, the dew point depression was expected to be greater for droplets with a diameter of 4 μm. This issue can be explained based on the entrainment effect. For droplets with a diameter of 4 μm, the entrainment value is zero (calculated by CFD modeling). However, for droplets with a diameter of 2 μm, the entrainment value is greater than 0. Therefore, smaller liquid droplets escaped from the 3S. This escape of liquid droplets caused less displacement of the dew point line for smaller droplets. In this section, the effect of employing integrated configuration on the PED was also investigated. As can be seen (Fig. 11), in the integration process, the displacement of the dew point curve of the product was higher than the JT process. It is evident that the greater the displacement of the dew point line, the greater the NGL recovery rate. Consequently, the conducted simulations illustrated that the new proposed processes (configuration B and C) scheme provided higher NGL recovery rate for a specified operating condition. The integrated processes can separate both heavy HCs and light HCs using the JT process and 3S process, respectively. Based on the PED, for configuration B after heavy HCs separation in the first stage, the lighter HCs will be separated by second stage processes. Separating lighter HCs, such as ethane and propane, was also improved in the integrated processes (configuration B and C) compared to the two stages JT process (configuration A). Therefore, the NGL recovery rate and dew point depression were improved for the integrated process compared to the JT process.

A comparison of Figs. 10 and 11 showed that in the JT process, separation of liquids occurred at the outlet of the JT valves. However, in the 3S process, this separation occurred at the outlet of the Laval nozzle. It is evident that the outlet of the Laval nozzle has a much lower temperature than the outlet of the 3S. Therefore, in the JT process, the PED of the outlet gas corresponds to the outlet of the JT valve. However, for the 3S process, the PED of the outlet gas corresponds to the outlet of the Laval nozzle. Of course, this conclusion is correct when the entrainment value is zero. Therefore, it should be noted that for cases where the entrainment is greater than zero, the behavior is completely different. Therefore, while the outlet gas from the JT process is always saturated (Fig. 10A–D), the outlet gas from 3Ss can include all three modes: saturated, sub-saturated, and super-saturated. These modes can be easily detected by comparing the outlet PED and the outlet operating conditions. Based on the simulations performed in Section “Optimization of swirler structure”, for the liquid droplets with a diameter greater than 4 μm, the entrainment value is always zero and as a result the state of the outlet gas from the 3S is always sub-saturated. However, for droplets with a diameter of 2 μm, the entrainment value is always greater than zero, and as a result, the exhaust gas state can be sub-saturated, saturated or supersaturated. Therefore, in order to properly understand the behavior of the 3S, the entrainment through 3S should be considered.

**Figure 12:**
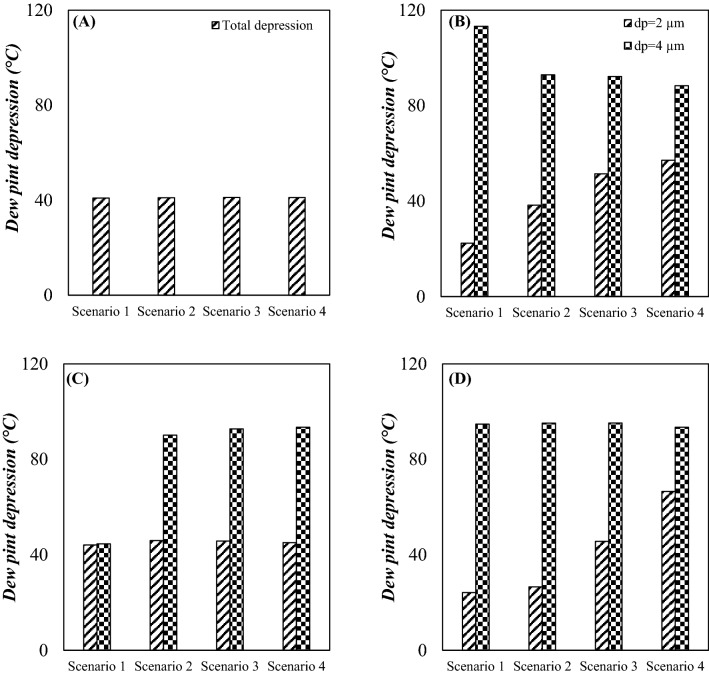
Total dew point depression for various scenarios and configurations: (**A**) configuration (**A, B**) configuration (**B, C**) configuration (**C** and **D**) configuration (**D**).

The produced natural gas from underground should be treated to correct its HCDP and achieve the sale gas specification. In this section, the condensation properties of natural gas were analyzed and the dew point depression for each separation method were reported. The HCDP highly depends on natural gas composition. Therefore, by liquefaction and separation of heavy HCs the HCDP vary significantly. The values of dew point depression (ΔT_d_) for various defined scenarios are presented in Fig. 12. It can be observed that the intermediate pressure ratio, process scheme and type of separation equipment have a significant influence on the HCDP of outlet gas. Furthermore, the simulation results demonstrated that the 3S was more efficient than the JT valve in decreasing the HCDP and improving the NGL recovery rate. Based on the simulation results, in the JT process (configuration A), the highest achievable HCDP depression is about 41.1 °C, which means that a high amount of heavy HCs escaped along with the product gas without being separated. Contrary to this, as shown in Fig. 12, for the integrated process (configuration B and C) and 3S process (configuration D), the maximum dew point depression was about 113.2 °C and 95.2 °C, respectively. Therefore, the configuration A with the maximum dew point depression of 41.1 °C and configuration B with the maximum dew point depression of 113.2 °C, showed the highest and lowest HCDP depression among the considered cases, respectively. Furthermore, for various configurations, the influence of variation of intermediate pressure ratio on dew point depression were also investigated. It can be observed that by increasing the PLR, the dew point depression was increased. Therefore, the dew point depression can be improved through modification of gas plant scheme and optimization of the operating condition. In addition, as shown in Fig. 12, the droplet size has a significant effect on the HCDP depression. According to this figure, the tiny droplets were less influenced by the induced centrifugal force and hardly directed toward the separator wall. Therefore, smaller liquid droplets were entrained by the continuous phase toward the separator outlet, which deteriorated the dew point depression.

Although, for 4 μm liquid droplets (Figs. 8 and 12), there is a direct relationship between the NGL recovery rate and the dew point depression, for 2 μm diameter droplets, this behavior was completely different. An important point from the comparison between Figs. 8 and 12 is that the NGL recovery rate and the dew point depression were not necessarily directly related. In other words, the highest HCDP depression did not necessarily cause the highest NGL recovery rate. In some cases, completely opposite behavior was observed. For example, for configuration B (d_p_ = 2 µm), the highest NGL recovery rate was obtained for scenario 1, however this scenario showed the lowest dew point depression. This difference in behavior was due to differences in the droplet size and entrainment effect. Therefore, only in cases where the entrainment value was zero, with further dew point depression, a higher NGL recovery rate was obtained. Contrary to this, for cases where the entrainment value is greater than zero, the relationship between NGL recovery rate and the dew point depression was completely different. Therefore, the dew point depression measurement method is not a suitable method to evaluate the performance of a gas plant. For example, although for scenario 1 of configuration A, the dew point depression was much greater than for scenario 1 of configuration D (d_p_ = 2 µm), as the latter was associated with about 234.1% higher NGL recovery rate.

**Figure 13:**
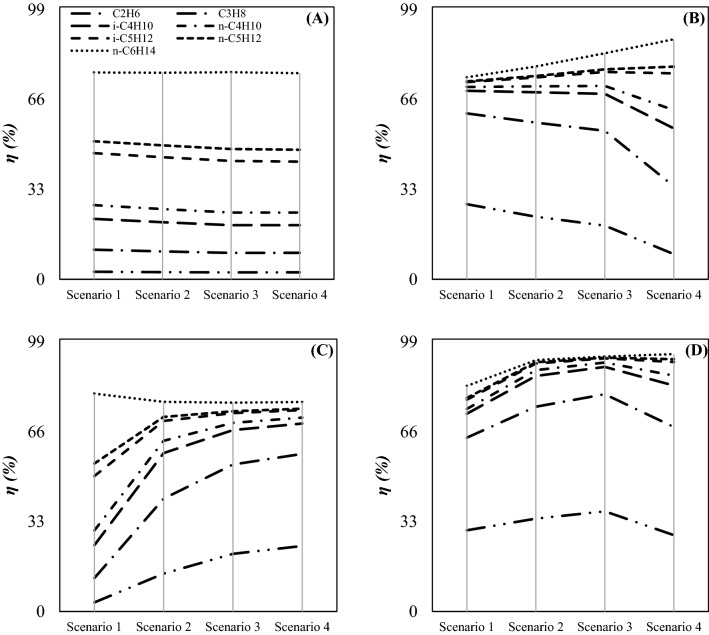
Component collection efficiency for various scenarios and configurations: (**A–D**) correspond to configurations (**A–D**) respectively with dp = 2 µm.

Another method that can be used to characterize and compare the capabilities of the different separation methods is the determination of component collection efficiency. The mole fraction of each component at the inlet and outlet of the 3S showed the separation capability of the 3S. Figure 13 shows the influence of installing 3S process instead of JT process on the component collection efficiency of light and heavy HCs for droplet diameter of 2 µm. The simulation results presented in Fig. 13 demonstrated that by replacing the 3S process with the JT process, the mole fraction of lighter HCs in the product decreased significantly. In addition, it can be seen that for all of the investigated configurations, the mole fraction of heavier components like hexane were negligible compared to the feed gas. Therefore, all of the investigated configurations showed appropriate separation efficiency for heavy HCs. However, JT process (configuration A) did not show good separation efficiency for light HCs. Consequently, to recover light HCs, much lower refrigeration temperature was required compared to the available minimum temperature by the JT process (configuration A). Therefore, the 3S process (configuration D) was in this respect superior to JT process (configuration A). This can be explained based on the mole fraction of lighter HCs at the outlet. For example, for an identical operational condition (for d_p_ = 2 µm), the highest propane recovery was about 10.86% and 79.75% for JT process (configuration A) and 3S process (configuration D), respectively. In conclusion, the 3S process is appropriate for the separation of lighter and heavier HCs. However, the JT process is preferred for the separation of heavier HCs.

The separation efficiency of different configurations was completely different for various scenarios and components. According to Fig. 13, configuration A showed a fixed component collection efficiency for different scenarios, while configuration D showed a higher component collection efficiency for scenario 3. For example, for configuration D the ethane collection efficiency was improved from 29.8% to 36.7% by increasing the intermediate pressure ratio from 0.27 to 1.5 (for d_p_ = 2 µm). For configuration B, with increasing the intermediate pressure ratio (scenario 1 to 4), the separation efficiency of light and heavy components decreased and increased, respectively (Fig. 13). The reason for this behavior was that by going from scenario 1 to scenario 4, the pressure gradient in the 3S decreased, and in the JT valve increased (Table 1), respectively. Therefore, by decreasing the pressure gradient in the 3S, due to the decreasing in the cooling depth, the lighter HCs did not separate well. However, by increasing the pressure gradient in the JT valve, the heavy HCs were better separated.

**Figure 14:**
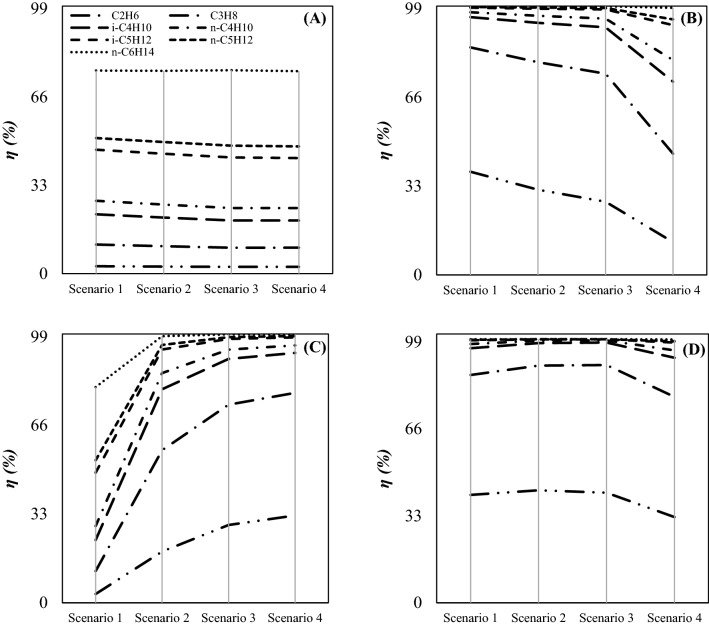
Component collection efficiency for various scenarios and configurations: (**A–D**) correspond to configurations (**A–D**) respectively with dp = 4 µm.

Although, the component collection efficiency of 4 μm droplets was higher than that of 2 μm droplets. A comparison of Figs. 13 and 14 showed that the component collection efficiency for these droplet sizes showed the same behavior. For example, when the liquid droplet size was 4 µm, the highest ethane collection efficiency was about 42.68%. However, when the size of liquid droplets decreased to 2 µm, the highest ethane collection efficiency reduced to 36.7%. This behavior can be explained based on the role of centrifugal force. Although, for tiny droplets, the applied centrifugal force was small, while for large droplets it was relatively high. Therefore, with increasing the liquid droplet size, the component collection efficiency increased.

### Exergy efficiency

**Figure 15:**
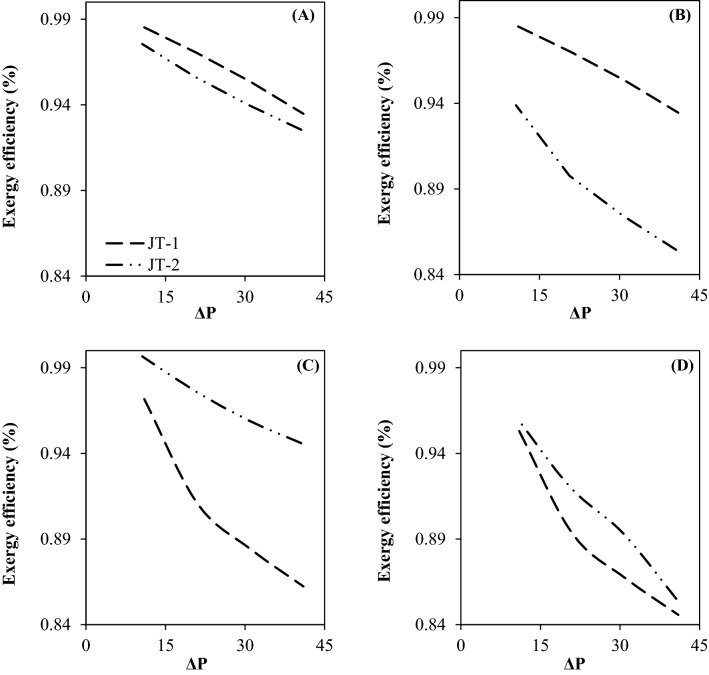
Exergy efficiency for various investigated configuration as function of pressure gradient: (**A**) configuration (**A, B**) configuration (**B, C**) configuration (**C** and **D**) configuration (**D**).

In this section, the exergy analysis was used to compare the performance of the JT process with the 3S process. Exergy analysis can be employed to optimize a specified chemical process. By exergy analysis, the amount of energy loss can be determined for each process. Figure 15 shows the exergy efficiency of various configurations in terms of pressure gradient. The JT process (configuration A) showed higher exergy efficiency when compared to the 3S processes (configuration D) and integrated processes (configuration B and C). Therefore, at similar operating conditions, the JT process (configuration A) provided the highest exergy efficiency between investigated processes. Contrary to this, the 3S process (configuration D) provided the lowest exergy efficiency. In addition, Fig. 15 provides a comparison of exergy efficiency of the second and third stage separation process. It can be observed that the pressure gradient had a significant influence on the exergy efficiency of each stage. Generally, for both of the JT and the 3S process, the highest exergy efficiency was obtained for the lowest pressure gradient. Furthermore, the simulation results and the exergy analysis demonstrated that for a specified pressure gradient, with replacing the 3S with the JT process, the exergy efficiency was decreased significantly. This behavior is due to the higher NGL recovery rate for the 3S process than the JT process. Therefore, part of the system's exergy was removed by the separated NGL. This issue resulted in a decrease of the system's exergy efficiency. Consequently, although the use of 3S instead of JT process significantly improved the NGL recovery rate, this modification simultaneously reduced the exergy efficiency.

**Figure 16:**
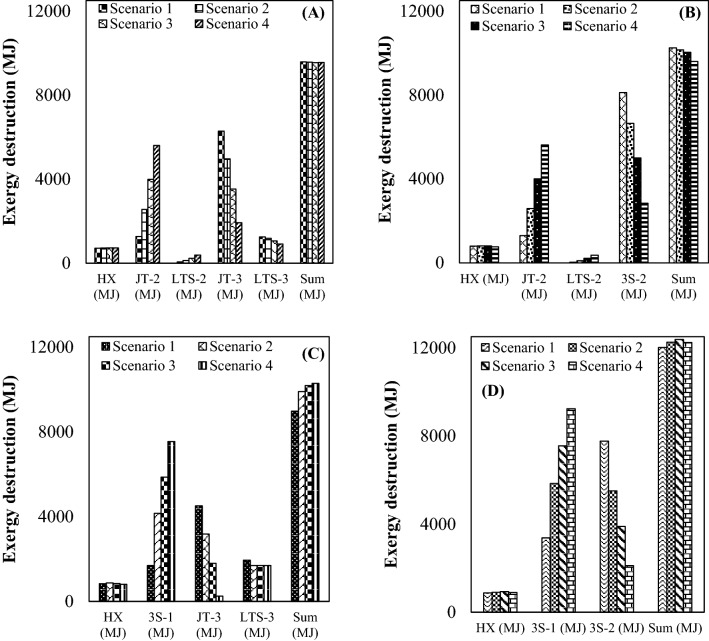
Exergy destruction for various investigated configuration as function of pressure gradient: (**A–D**) correspond to configurations (**A–D**) respectively.

The total exergy destruction of a gas plant was defined as the sum of the exergy destruction by various process equipment such as valve, HX and separator. The exergy destruction is due to several reasons including: pressure drop inside the 3S and JT valve, temperature difference through the HX, energy loss and friction through the system. The exergy destruction is a direct consequence of system irreversibility. Figure 16 shows the distribution of exergy destruction for various configurations, where the contribution of each equipment in the total exergy destruction is presented. The results of exergy analysis showed that the JT valve and the 3S had the highest exergy destruction compared to other process equipment. Furthermore, configuration A showed lower exergy destruction compared to configuration D. Moreover, the exergy destruction of integrated configurations (configuration B and C) was also higher than the JT process (configuration A). Therefore, modification of the process scheme influenced the amount of exergy destruction. In addition, by comparing Figs. 8 and 16, it can be seen that in those scenarios where the NGL recovery rate was higher, the exergy destruction was also higher. Consequently, to increase the NGL recovery rate, more pressure energy should be consumed. In addition, it should be noted that by modifying the configuration of the JT process (configuration A), the maximum amount of exergy destruction increased about 29.6%. However, this structural modification (scenario 3 in configuration D) increased the NGL recovery rate by at least 323% compared to the JT process (scenario 3 in configuration A). Therefore, although the structural modification increased the exergy destruction, the improvement in the NGL recovery rate was much more significant.

### Optimal operating point of the studied gas plant

**Figure 17:**
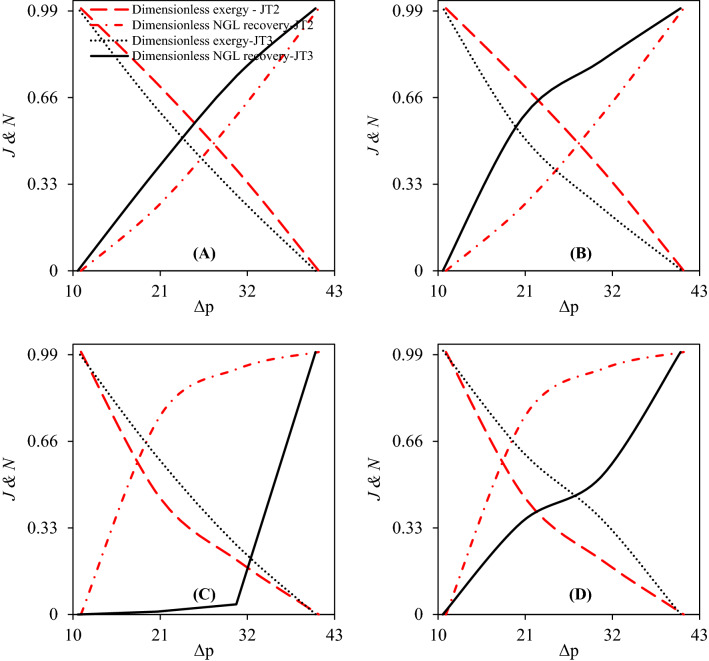
Optimization of investigated gas plant based on the dimensionless exergy efficiency and NGL recovery rate: (**A–D**) correspond to configurations (**A–D**) respectively.

This section focused on the optimization of operational condition for various configurations according to the NGL recovery rate and exergy efficiency. With the increase of the pressure gradient through each stage, the NGL recovery rate significantly increased. Contrary to this, the exergy efficiency of that stage considerably decreased. Consequently, there is a tradeoff between the NGL recovery rate and exergy efficiency. Accordingly, in order to find the optimal operating point, two dimensionless parameters including dimensionless exergy efficiency and dimensionless NGL recovery rate were defined as follow:

28 $$ J = \frac{{\varepsilon - \varepsilon_{Minimum} }}{{\varepsilon_{Maximum} - \varepsilon_{Minimum} }} $$

29 $$ N = \frac{{NGL - NGL_{Minimum} }}{{NGL_{Maximum} - NGL_{Minimum} }} $$

where *J* and *N* are the dimension exergy efficiency and dimensionless NGL recovery rate. Figure 17 shows the influence of the pressure gradient at each stage on the dimensionless exergy efficiency and dimensionless NGL recovery rate of configurations A, B, C and D. The simulation result demonstrated that the pressure gradient at each stage had a significant influence on these dimensionless parameters. According to Fig. 17, the dimensionless NGL recovery rate increased and dimensionless exergy efficiency decreased by increasing the pressure gradient at each stage. The point of interference between dimensionless exergy efficiency and dimensionless NGL recovery rate indicated the optimal operating point for each configuration. As can be seen in Fig. 17, for configuration A and B, in order to achieve optimal operating conditions, the pressure drop in the second stage must be greater than in the third stage. Contrary to this, for configurations C and D, the pressure drop in the third stage must be greater than in the second stage.

## Conclusion

This study compares the exergy efficiency and natural gas liquid recovery rate of a gas plant for different operating conditions and process schemes. It was observed that by employing the supersonic separator process instead of the Joule–Thomson process, the natural gas liquid recovery rate as well as energy consumption increased significantly. The simulation results showed that the maximum natural gas liquid recovery rate of the Joule–Thomson process (configuration A) was about 222 kmol/h while this value was about 1088.4 kmol/h (for the liquid droplets with diameter of 4 µm) in the supersonic separator process (configuration D). Therefore, by replacing the supersonic separator process with the Joule–Thomson process, the efficiency of the system was significantly improved. In addition, separation of light hydrocarbons like ethane from the natural gas can be performed by the supersonic separator process. However, the Joule–Thomson process can only separate the heavier hydrocarbons. The effect of operational parameters on the system performance was also investigated. Although, it was observed that the optimal operating point for each configuration is different from the other. Generally, at the intermediate pressure ratio the optimal exergy efficiency and natural gas liquid recovery rate was obtained. Simulation results demonstrated that larger pressure gradients provided higher natural gas liquid recovery rate and lower exergy efficiency. Furthermore, the Joule–Thomson valves and supersonic separator are the main source of exergy destruction in the investigated gas plant.

The phase envelope diagram and the amount of dew point depression were also investigated for various operating conditions and process schemes. It was observed that at the optimal condition (the entrainment value equal to zero) the dew point of the dry gas for the supersonic separator process (configuration D) was 54.1 °C lower than the Joule–Thomson process (configuration A). Furthermore, it was observed that by integration of the Joule–Thomson with the supersonic separator process, the production rate and the dew point depression was improved significantly. In addition, the simulation results showed that the phase envelope diagram and the dew point depression are a strong function of the liquid droplet size. It was shown that the liquid droplets of 4 µm are completely separated from the continuous phase. Therefore, for larger droplets the dew point depression is higher. Furthermore, the liquid droplets cannot be separated completely when their diameter is about 2 µm. This phenomenon caused that when the entrainment value was greater than zero (for droplet diameter of 2 µm), measuring the dew point was not an appropriate criterion to evaluate system performance. Therefore, in cases where the entrainment value was greater than zero, it is better to evaluate the system performance by measuring the natural gas liquid recovery rate instead of measuring the dew point depression.

## Data Availability

The datasets used and/or analysed during the current study available from the corresponding author on reasonable request.

## List of symbols

C: Cooling performance
C_*μ*_: Constant (-)
C_*p*_: Heat capacity at constant pressure (Jkg^−^^1^ K^−^^1^)
C_*ε1*_: Constant (-)
C_*ε2*_: Constant (-)
E*x, e*: Exergy (J)
F_*s*_, *F*: Confidence factor, force (kg m^1^s^−^^2^)
H: Enthalpy (Jkg^−^^1^)
k: Turbulence kinetic energy (m^2^s^−^^2^)
L: Length (m)
m: Mass (kg)
N: Collection efficiency
P, *p*: Pressure (Pa), convergence order
Q: Heat source (Wm^−^^3^)
Q*p*: Pressure work (Wm^−^^3^)
R, *r*: Gas constant (Jmol^−^^1^ K^−^^1^), refinement rate, radius (m)
S: Separation efficiency, entropy (kg m^2^ s^− ^^2^ K^− ^^1^)
T, *t*: Temperature (K), time (s)
u, v, w: Gas velocity (ms^−^^1^)
V, *v*: Gas molar volume (m^3^mol^−^^1^)
W: Work (kg m^2^s^−^^2^)
x, y ,z: Axis coordinate (-)

## Subscripts

d: Destruction
In, Inlet: Inlet
JT: Joule–Thomson coefficient
m: Minimum
out: Outlet
Ref: Reference
th: Throat

## Greek letters

α: Elliptic blending function
ζ: Turbulent relative fluctuations
ε: Turbulence dissipation rate (m^2^s^−^^3^), exergy efficiency (-)
k: Heat conduction (Wm^−^^1^ K^−^^1^)
τ: Viscous stress tensor (Pa)
µ: Gas dynamic viscosity (Pa.s)
μ_*T*_: Turbulence viscosity (Pa.s)
μ: Joule–Thomson coefficient
ρ: Gas density (kgm-3)
σ_*k*_: Turbulence Prandtl number (-)
σ_*ε*_: Turbulence Prandtl number (-)
$\phi$: Viscous dissipation term
φ: Divergence angle (°)
θ: Vane angle (°)

## Abbreviations

CFD: Computational fluid dynamic
DP: Dew point
EOS: Equation of state
GCI: Grid convergence index
HC: Hydrocarbon
HX: Heat exchanger
JT: Joule–Thomson
JTC: Joule–Thomson coefficient
LTS: Low temperature separator
NGL: Natural gas liquid
PED: Phase envelope diagram
PLR: Pressure loss ratio
PR: Peng-Robinson
3S: Supersonic separator
SRK: Soave–Redlich–Kwong
